# Ultra-sensitivity in reconstructed exceptional systems

**DOI:** 10.1093/nsr/nwae278

**Published:** 2024-08-16

**Authors:** Tian Chen, Deyuan Zou, Zilong Zhou, Ruiguo Wang, Yue Feng, Houjun Sun, Xiangdong Zhang

**Affiliations:** Key Laboratory of Advanced Optoelectronic Quantum Architecture and Measurements of Ministry of Education, Beijing Key Laboratory of Nanophotonics & Ultrafine Optoelectronic Systems, and School of Physics, Beijing Institute of Technology, Beijing 100081, China; Key Laboratory of Advanced Optoelectronic Quantum Architecture and Measurements of Ministry of Education, Beijing Key Laboratory of Nanophotonics & Ultrafine Optoelectronic Systems, and School of Physics, Beijing Institute of Technology, Beijing 100081, China; School of Mechatronical Engineering, Beijing Institute of Technology, Beijing 100081, China; School of Mechatronical Engineering, Beijing Institute of Technology, Beijing 100081, China; School of Mechatronical Engineering, Beijing Institute of Technology, Beijing 100081, China; Beijing Key Laboratory of Millimeter Wave and Terahertz Techniques, and School of Information and Electronics, Beijing Institute of Technology, Beijing 100081, China; Key Laboratory of Advanced Optoelectronic Quantum Architecture and Measurements of Ministry of Education, Beijing Key Laboratory of Nanophotonics & Ultrafine Optoelectronic Systems, and School of Physics, Beijing Institute of Technology, Beijing 100081, China

**Keywords:** ultra-sensitivity sensor, non-Hermitian, reconstructed exceptional system, long-range coupling, circuit network

## Abstract

Sensors are of fundamental importance and widely used in modern society, such as in industry and environmental monitoring, biomedical sample ingredient analysis and wireless networks. Although numerous sensors have been developed, there is a continuous demand for sensors with increased sensitivity, to detect signals that were previously undetectable. Recently, non-Hermitian degeneracies, also known as exceptional points (EPs), have attracted attention as a way of improving the responsiveness of sensors. In contrast to previous investigations, here we present a new approach to achieving ultra-sensitivity by reconstructing exceptional systems. In the reconstruction process, some eigenstates near the previous EPs are utilized, and non-reciprocal long-range couplings are introduced. The sensitivities of our reconstructed systems have improved by several orders of magnitude compared to those based on EPs. Furthermore, we design and fabricate corresponding integrated circuit sensors to demonstrate the scheme. Our work paves the way for the development of highly sensitive sensors, which have a wide range of applications in various fields.

## Teaser

A reconstructed exceptional sensor with non-reciprocal long-range couplings is theoretically designed and experimentally implemented on a circuit network, where the sensor's sensitivity improves several orders of magnitude compared with exceptional-point sensors.

## INTRODUCTION

An exceptional point (EP) is a non-Hermitian degeneracy or branch point where eigenvalues and the corresponding eigenvectors coalesce [1–8]. For a system supporting an *N*th-order EP, the reaction to a perturbation ($\varepsilon $) is expected to follow an *N*th-root behavior (${{\varepsilon }^{{\mathrm{1/}}N}}$) [9–24]. This is in contrast with Hermitian systems, where the sensing response is at best of order $\varepsilon $. This opens up the possibility of designing an ultra-sensitive sensor based on such a non-Hermitian spectral singularity. In recent years, there have been advancements in EP-based sensor design, leading to increased sensor responsivities as demonstrated in studies [10–24]. However, the signal-to-noise performance of such sensors has been the subject of controversial debate in recent studies [25–32]. That is, the signal-to-noise-ratio (SNR) at EPs is proportional to the perturbation, which hinders the enhancement of detection precision for EP-based sensors. Fortunately, recent investigations have shown that the problems can be overcome by developing some effective methods [20,21,33–37]. In particular, it has been proven that the designed parity-time-symmetric sensing circuit platform with EPs can offer combined enhanced sensitivity, improved resolution and non-degraded thermal noise performance, providing an exciting prospect for next-generation sensing technologies [13,16,20]. The concept of ‘sensitivity’ in our work is used to estimate the transduction coefficient of the sensor from the input quantity to the output quantity.

Here, we propose a new method of achieving ultra-sensitivity through the theoretical reconstruction and experimental demonstration of reconstructing exceptional systems. By considering the non-Hermitian system with the *N*th-order EP, we solve the corresponding eigenstates and use them in the expansion to obtain the eigenstates of the system near the EP. Then, we employ some of these eigenstates to construct a new system called a reconstructed exceptional system. In the reconstruction process, non-reciprocal long-range couplings are introduced, and the coupling strengths exhibit a power-law relationship with the length of the chain. It is found that the sensitivities of the reconstructed systems have improved by several orders of magnitude compared to the previous EP systems. The corresponding integrated circuit sensors are designed and fabricated to demonstrate the scheme. Thus, our work opens up the exciting possibility of realizing sensors with unprecedented sensitivity.

## RESULTS

### Theory of ultra-sensitivity in reconstructed exceptional systems

**Figure 1.**
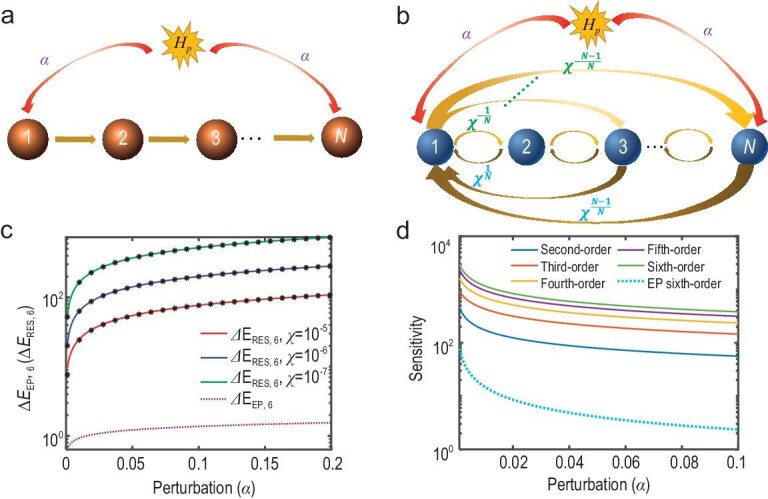
EP and reconstructed exceptional system for the sensor. (a) The system possessing the *N*th-order EP. (b) The reconstructed exceptional system. The non-reciprocal coupling strengths increase with the distance between lattices. In (a) and (b), the surrounding perturbation only acts on the first and last lattice of the system, and the coupling strength is $\alpha $. (c) The relationship between energy difference and perturbation strength $\alpha $ for the sixth-order EP system and the sixth-order reconstructed exceptional system. The top three solid lines represent the cases with $\chi = {{10}^{ - 5}}$, ${{10}^{ - 6}}$ and ${{10}^{ - 7}}$, respectively. (d) The sensitivity $\frac{{\partial \Delta {{E}_{{\mathrm{RES,}}N}}}}{{\partial \alpha }}$($\frac{{\partial \Delta {{E}_{{\mathrm{EP}},6}}}}{{\partial \alpha }}$) with perturbation strength $\alpha $. The top five solid lines from top to bottom correspond to the reconstructed exceptional system with *N* from 6 to 2. The dotted line at the bottom denotes the sensitivity in the sixth-order EP system.

We consider the 1D lattice model as shown in Fig. 1a. It consists of *N* lattices, which are represented by orange spheres from 1 to *N*. The arrows denote the non-reciprocal couplings between the nearest-neighboring lattices. When the coupling strengths are taken as 1 from left to right and 0 from right to left, the whole lattice model forms a non-Hermitian system possessing the *N*th-order EP. That is, the eigen-equation of the system is expressed as ${{H}_{\mathrm{0}}}| {{{\psi }_{\mathrm{0}}}} \rangle {\mathrm{\, =\, }}{{\varepsilon }_{\mathrm{0}}}| {{{\psi }_{\mathrm{0}}}} \rangle $. Here, ${{H}_{\mathrm{0}}}$, $| {{{\psi }_{\mathrm{0}}}} \rangle $ and ${{\varepsilon }_{\mathrm{0}}}$ represent the system Hamiltonian, eigenstate and eigenvalue, respectively. When solving the eigen-equation, not only *N* eigenvalues but also the corresponding *N* states coalesce. Such coalescence of eigenstates results in high sensitivity when this *N*th-order EP system is applied as a sensor to detect surrounding perturbations. As shown in Fig. 1a, when adding the surrounding perturbation ${{H}_p}$, the relation between the energy dispersion and the perturbation strength $\alpha $ is $\Delta {{E}_{{\mathrm{EP}},N}} \approx {{\alpha }^{\frac{1}{N}}}$, and the sensitivity for this EP system is $\frac{{\partial \Delta {{E}_{{\mathrm{EP}},N}}}}{{\partial \alpha }} \approx \frac{1}{{{{\alpha }^{\frac{{N - 1}}{N}}}}}$ [9–11,24]. Therefore, a tiny coupling from the perturbation can cause a significant change in energy difference between two neighboring eigenstates. In this way, the EP system can realize the high-precision sense for a tiny perturbation [9–11,24].

Here, we design a new non-Hermitian system that builds on the previous EP system. A rather small perturbation is added to ${{H}_{\mathrm{0}}}$ as $\chi {{H}_1}$, in which the value of $\chi $ is small and represents the extent of deviation from the *N*th-order EP, and ${{H}_{\mathrm{1}}}$ denotes a linear perturbation of ${{H}_0}$. In such a case, the system Hamiltonian is expressed as $H{\mathrm{\,=\, }}{{H}_0} + \chi {{H}_1}$. The eigen-equation is $H| \psi \rangle {\mathrm{ \,=\, }}\varepsilon | \psi \rangle $, where $\varepsilon $ is the eigenvalue and $| \psi \rangle $ is the corresponding eigenstate. If $\varepsilon $ and $| \psi \rangle $ follow the expansion in the fractional power series of the parameter $\chi $ (Newton-Puiseux series), they can be expressed as $\varepsilon {\mathrm{ \,=\, }}{{\varepsilon }_{\mathrm{0}}}{\mathrm{ + }}{{\chi }^{\frac{{\mathrm{1}}}{N}}}{{\varepsilon }_1} + {{\chi }^{\frac{2}{N}}}{{\varepsilon }_2} + \cdots + {{\chi }^{\frac{n}{N}}}{{\varepsilon }_n}$ and $| \psi \rangle {\mathrm{ \,=\, }}| {{{\psi }_{\mathrm{0}}}} \rangle {\mathrm{ + }}{{\chi }^{\frac{{\mathrm{1}}}{N}}}| {{{\psi }_1}} \rangle + {{\chi }^{\frac{2}{N}}}| {{{\psi }_2}} \rangle + \cdots + {{\chi }^{\frac{n}{N}}}| {{{\psi }_n}} \rangle $ [38]. By substituting the expansions of $\varepsilon $ and $| \psi \rangle $ into the eigen-equation, we can determine the exact forms of $| {{{\psi }_i}} \rangle \ $and ${{\varepsilon }_i}$  $( {1 \le i \le N - 1} )$. Details can be found in Note S1. For convenience, we choose the perturbation ${{H}_{\mathrm{1}}}$ to act on the first and last lattices of the system. In this case, we can obtain ${{\varepsilon }_1} = 1$ and ${{\varepsilon }_{i \ne 1}} = 0$. $| {{{\psi }_i}} \rangle \ $can be expressed as $| {{{\psi }_i}} \rangle = {{( {0,...,0,1,0,...,0} )}^T}$. Here the superscript *T* represents the transpose. That is, the $i + 1$th element in the column vector $| {{{\psi }_i}} \rangle $ is 1 and the others are zero. So, the eigenstate is expressed as $| \psi \rangle = {{( {1,{{\chi }^{\frac{{\mathrm{1}}}{N}}},{{\chi }^{\frac{2}{N}}}, \cdots ,{{\chi }^{\frac{{N - 1}}{N}}}} )}^T}$. In the same way, we can obtain the eigenstate for ${{H}^T}$ as $| \phi \rangle = {{( {{{\chi }^{\frac{{N - 1}}{N}}}, \cdots ,{{\chi }^{\frac{{\mathrm{2}}}{N}}},{{\chi }^{\frac{{\mathrm{1}}}{N}}},1} )}^T}$. Combing these two eigenstates, the Hamiltonian of the reconstructed exceptional system (RES), ${{H}_{{\mathrm{RES}},N}}{\mathrm{ \,=\, }}{{\chi }^{\frac{N}{{N - 1}}}}| \psi \rangle \langle \phi |$, can be designed. It is expressed as


(1) \begin{eqnarray*}
{\!\!{H}_{{\mathrm{RES}},N}} = {{\left( {\begin{array}{@{}*{4}{c}@{}} {\mathrm{1}}&{{{\chi }^{ - \frac{{\mathrm{1}}}{N}}}}& \cdots &{{{\chi }^{ - \frac{{N - 1}}{N}}}}\\
{{{\chi }^{\frac{{\mathrm{1}}}{N}}}}&1& \cdots &{{{\chi }^{ - \frac{{N - 2}}{N}}}}\\
\vdots & \vdots & \ddots & \vdots \\ {{{\chi }^{\frac{{N - 1}}{N}}}}& \cdots &{{{\chi }^{\frac{{\mathrm{1}}}{N}}}}&1 \end{array}} \right)}_{N \times N}}.\end{eqnarray*}

Detailed constructions can be found in Methods. The Hamiltonian ${{H}_{{\mathrm{RES}},N}}$ can be characterized by a 1D lattice model, as shown in Fig. 1b. Here, the spheres represent different lattices from 1 to *N*. The arrows pointing to the right denote forward couplings from the left to right lattice, and the arrows pointing to the left represent backward couplings from the right to left lattice. The forward coupling strengths are not equal to the backward couplings, and the non-reciprocal couplings in the lattice are established in this way. Unlike the EP system, the couplings of ${{H}_{{\mathrm{RES}},N}}$ not only exist in the nearest neighboring lattices, but also among the two distant lattices. The coupling strengths and the length of the chain exhibit a power-law relationship. For this reconstructed exceptional system, when adding the same perturbation ${{H}_p}$, the energy dispersion relation with the perturbation strength $\alpha $ can be calculated as $\Delta {{E}_{{\mathrm{RES}},N}} \approx {{\chi }^{ - ( {\frac{{N - {\mathrm{1}}}}{{2N}}} )}}{{\alpha }^{\frac{1}{2}}}$, and the sensitivity can be obtained as $\frac{{\partial \Delta {{E}_{{\mathrm{RES}},N}}}}{{\partial \alpha }} \approx \frac{1}{{{{\chi }^{\frac{{N - 1}}{{2N}}}}{{\alpha }^{\frac{1}{2}}}}}$. Details of the derivation can be found in Methods. When compared to the sensitivity of the EP-based sensor, it is found that the parameter $\chi $ is added in the expression of sensitivity for the reconstructed exceptional system. This means that we can realize very high-precision sensitivity by controlling $\chi $. Figure 1c displays a comparison of $\Delta {{E}_{{\mathrm{RES}},N}}$ between the reconstructed exceptional systems and the previous EP system. The dotted-dashed line corresponds to the energy difference $\Delta {{E}_{{\mathrm{EP}},N}}$ of the previous *N*th-order EP system. The other three solid lines represent the numerical results of $\Delta {{E}_{{\mathrm{RES}},N}}$ for the *N*th-order reconstructed exceptional system with $\chi = {{10}^{ - 5}}$, ${{10}^{ - 6}}$ and ${{10}^{ - 7}}$, respectively. The dots denote the calculated results from the analytical equation $\Delta {{E}_{{\mathrm{RES}},N}} \approx {{\chi }^{ - ( {\frac{{N - {\mathrm{1}}}}{{2N}}} )}}{{\alpha }^{\frac{1}{2}}}$. It can be found that the numerical results and the analytical results for $\Delta {{E}_{{\mathrm{RES}},N}}$ agree with each other very well. In Fig. 1c, it is clearly seen that the energy difference $\Delta {{E}_{{\mathrm{RES}},N}}$ between the neighboring eigenstates in the reconstructed exceptional system is two orders of magnitude larger than that in the EP system.

In addition, the sensitivity results are also provided. In Fig. 1d, we provide the sensitivity for the RES with the order N ranging from 2 to 6, and the corresponding results are shown. It can be clearly seen that, with an increase in order N, the value of sensitivity also increases. Even for the reconstructed exceptional system with the order N = 2, its sensitivity (solid line) is also two orders of magnitude larger than that of the sixth-order EP system (dotted line). As for the sixth-order RES, its sensitivity is at least three orders of magnitude larger than the corresponding sixth-order EP system. Moreover, the sensitivity of the higher-order (N > 6) RES is better than that of the sixth-order RES. Details are provided in Note S2. The enhanced sensitivity in this reconstructed exceptional system can not only be analyzed in the theoretical frame, but also studied in experimental platforms for realistic application. In the following, we explore how to implement the above scheme in an electric circuit platform [39–49] for the enhanced detection of displacement.

### Ultra-sensitive circuit sensors based on reconstructed exceptional systems

**Figure 2.**
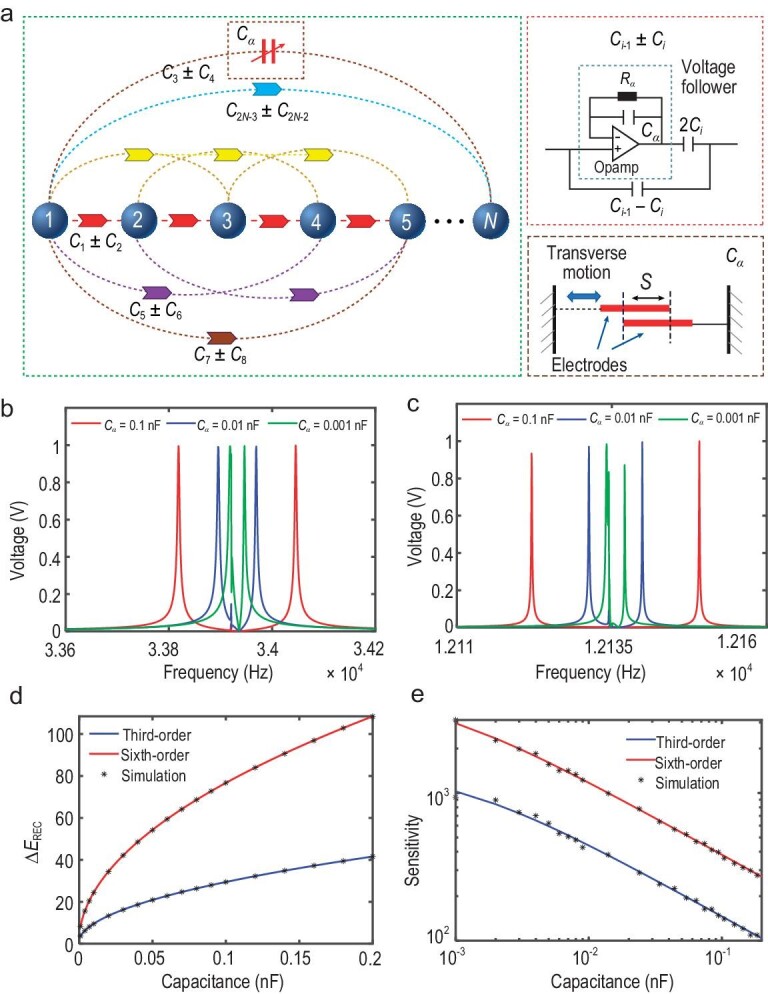
Circuit design and results for ultra-sensitivity. (a) Illustrations of a classical circuit for ultra-sensitivity with *N* nodes. Arrows represent non-reciprocal capacitors with varying values. Right: the dashed box at the top shows the internal structure of the non-reciprocal capacitor, and the dashed box at the bottom shows the diagram of variable capacitor ${{C}_\alpha }$, whose value is controlled by the relative motion of two electrodes. (b) and (c) show the resonance frequencies in the third- and sixth-order RECs, respectively. In these two panels, solid lines represent the cases with different values of ${{C}_\alpha }$, which are 0.1, 0.01 and 0.001 nF, respectively. (d) The analytical and simulation results for the splitting of resonance frequency in RECs. The analytical results for the third- and sixth-order RECs are shown as solid lines, respectively. The corresponding electrical simulation results are shown as dots. (e) The analytical and simulation results for sensitivity in the RECs. The analytical results for the third- and sixth-order RECs are shown as solid lines, respectively. The electrical simulation results are shown as dots.

Now, we discuss how to design circuit sensors based on reconstructed exceptional systems. The theoretical model of our designed sensor is schematically shown in Fig. 2a. Corresponding to Fig. 1b, the circuit in Fig. 2a also includes *N* nodes marked by spheres. The connections between nodes for the non-reciprocal coupling are achieved by non-reciprocal capacitors marked with different arrows. These arrows represent different values of capacitors ${{C}_{i - 1}} \pm {{C}_i}$ ($i = 2,4,...,2N - 2$). The design of the non-reciprocal capacitor leads to the non-reciprocal connections between circuit nodes. Following this design, the value from the left node to the right one is ${{C}_{i - 1}} + {{C}_i}$, and from the right node to the left one is ${{C}_{i - 1}} - {{C}_i}$. For example, the nearest-neighbor connection from node 1 to 2 is ${{C}_1} + {{C}_2}$, and from node 2 to 1 is ${{C}_1} - {{C}_2}$. In Note S3, we give details of the working function of the non-reciprocal capacitor. Here, by designing the value of capacitance, we can realize the coupling strength with the power-law relationship. The power-law relationships of coupling strengths in Equation (1) are ${{\chi }^{ - \frac{{\mathrm{1}}}{N}}},{{\chi }^{ - \frac{2}{N}}}, \cdots ,{{\chi }^{ - \frac{{N - 1}}{N}}}$ and ${{\chi }^{\frac{{\mathrm{1}}}{N}}},{{\chi }^{\frac{2}{N}}}, \cdots ,{{\chi }^{\frac{{N - 1}}{N}}}$. When we choose the values of capacitors to satisfy ${{C}_{i - 1}} + {{C}_i}{\mathrm{ \,=\, }}{{\chi }^{ - i/2N}}$and ${{C}_{i - 1}} - {{C}_i}{\mathrm{ \,=\, }}{{\chi }^{i/N}}$($i = 2,4,...,2N - 2$), the non-reciprocal coupling relations in Fig. 1b can be achieved in our designed circuit networks.

In addition, to implement the corresponding diagonal terms of the Hamiltonian of Equation (1) in the circuit, each node should be connected to appropriate grounding elements to achieve the same on-site potentials. Details of the grounding elements are provided in Note S4. For such a circuit network, we can theoretically derive its circuit Laplacian *J* and prove that it corresponds to the Hamiltonian ${{H}_{{\mathrm{RES}},N}}$ of the lattice model described in Equation (1). Moreover, the resonance frequency in the circuit also has a one-to-one correspondence to the eigenvalue of the Hamiltonian ${{H}_{{\mathrm{RES}},N}}$. In Note S5, we give a demonstration of such a relation between the *J* and ${{H}_{{\mathrm{RES}},N}}$.

The perturbation ${{H}_p}$ in Fig. 1b can be realized by designing a variable capacitor ${{C}_\alpha }$, whose schematic diagram is shown in the dashed box at the top of Fig. 2a. The variable capacitor contains two electrodes, and its value can be varied by moving the two electrodes, relative to each other. By adjusting the value of the variable capacitor, different perturbations can be characterized. Due to the mentioned relation between the resonance frequency in the circuit and the eigenvalue of the Hamiltonian, the obtained resonance frequency at each ${{C}_\alpha }$ can be transformed to the eigenvalue ${{E}_{{\mathrm{REC}}}}$ of the reconstructed exceptional circuit (REC), which corresponds to the eigenvalue ${{E}_{{\mathrm{RES}}}}$ of the total Hamiltonian ${{H}_{{\mathrm{RES}},N}} + {{H}_P}$. Therefore, simulated results for the variation of the eigenvalue difference $\Delta {{E}_{{\mathrm{REC}}}}$ with a difference in perturbations ${{C}_\alpha }$ can be obtained. Furthermore, based on the eigenvalue difference $\Delta {{E}_{{\mathrm{REC}}}}$, we can determine the sensitivity $\frac{{\partial \Delta {{E}_{{\mathrm{REC}}}}}}{{\partial {{C}_\alpha }}}$ of our circuit.

In addition, the derivation of energy difference and sensitivity of the reconstructed exceptional system are not only analytically realized in quantum theory, we can also analytically derive the energy difference $\Delta {{E}_{{\mathrm{REC}}}} \approx {{( {{{C}_1} - {{C}_2}} )}^{ - ( {\frac{{N - {\mathrm{1}}}}{2}} )}}C_\alpha ^{\frac{1}{2}}$ and sensitivity $\frac{{\partial \Delta {{E}_{{\mathrm{REC}}}}}}{{\partial {{C}_\alpha }}} \approx \frac{1}{{{{{( {{{C}_1} - {{C}_2}} )}}^{\frac{{N - 1}}{2}}}{{C}_\alpha }^{\frac{1}{2}}}}$ in the circuit. They have a one-to-one correspondence with the energy difference and sensitivity of the RES. The detailed derivations are shown in Note S6. Therefore, the designed circuit network can implement an ultra-sensitive performance based on the RES above.

To evaluate the performance of our designed circuit network, we use the simulation software LTspice to calculate the voltages of the circuit systems with various orders. The distributions of voltage as a function of frequency for the third- and sixth-order RECs are provided in Fig. 2b and c, respectively. The parameters of capacitors are chosen in accordance with lattice models with $\chi {\mathrm{ \,=\, }}{{10}^{ - 5}}$. Three solid lines represent the distributions of voltage with perturbation capacitor values as ${{C}_\alpha } = 0.1$, 0.01 and 0.001 *nF*. As shown in Fig. 2b and c, different resonance frequencies appear for different ${{C}_\alpha }$. The derivations of these resonance frequencies are presented in Note S7. Even for a rather small perturbation with ${{C}_\alpha } = 0.001$*nF*, the two resonance peaks can be distinguished clearly. This means that our designed circuit sensors can indeed sense various small perturbations.

The energy difference $\Delta {{E}_{{\mathrm{REC}}}}$ and sensitivity $\frac{{\partial \Delta {{E}_{{\mathrm{REC}}}}}}{{\partial {{C}_\alpha }}}$ based on the resonance frequencies for the third- and sixth-order RECs are shown in Fig. 2d and e, respectively. The solid lines are the analytical results obtained from the calculation shown in Note S6. The dots are simulated results using LTspice. Both analytical and simulated results are identical, indicating the accuracy of our analytical derivations in the RECs. It is noteworthy that these analytically obtained sensitivities in the RECs are consistent with those shown in Fig. 1d, which means that the circuit sensors with the ultra-sensitivity are successfully implemented. As well as for the third- and sixth-order RECs, simulated results for the other order RECs also demonstrate ultra-sensitivity. These are given in Note S8. As a comparison, we also simulate the energy difference and sensitivity for the EP circuit. The results are also shown in Note S8. It can be clearly seen that within the range of 3 *pF* to 200 *pF*, the sensitivity of the REC is about two orders of magnitude larger than that of the EP circuit. Therefore, instead of using the EP circuit, it is more advantageous to use the REC in the detection of small perturbations.

**Figure 3.**
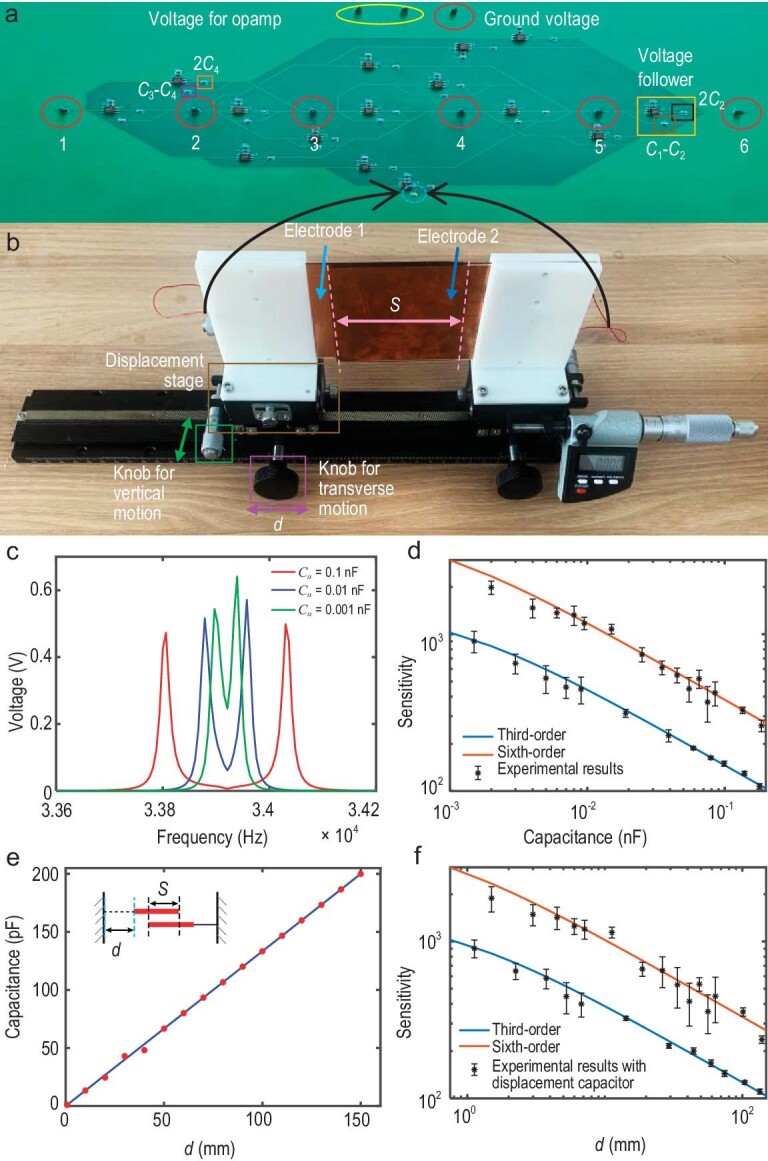
Experimental results regarding ultra-sensitivity. (a) A photograph of the fabricated sixth-order REC. The numbers from 1 to 6 are the nodes in the circuit. The capacitors connecting between different nodes in the circuit are shown. (b) A photograph of the displacement capacitor. The value of this variable capacitor is controlled by the relative motion between Electrode1 and 2. (c) The resonance frequencies in the third-order REC. Three solid lines represent the cases with different values of ${{C}_\alpha }$, which are 0.1, 0.01 and 0.001 nF, respectively. (d) Comparison of analytical and experimental results for sensitivity. Solid lines: analytical results of sensitivity from the third- and sixth-order RECs. Dots: experimental results of sensitivity in corresponding circuits. (e) The relationship between the displacement and the variation of capacitance in the displacement capacitor. The horizontal axis *d* denotes the distance from the left end to the left edge of Electrode1, which is illustrated in the inset. (f) Comparisons between theoretical and experimental results for sensitivity after adding the displacement capacitor. Solid lines: analytical results of sensitivity from the third- and sixth-order RECs. Dots: experimental results of sensitivity in RECs with the displacement capacitor.

Furthermore, we experimentally fabricate the circuit sensors described above. Figure 3a shows a photograph of the sixth-order REC. It contains six nodes marked by circles. Different non-reciprocal capacitors are marked by different rectangles, and the perturbation capacitor ${{C}_\alpha }$ is marked by the circle at the bottom of Fig. 3a. The fabricated circuit corresponds with the theoretical design shown in Fig. 2a. Detailed discussions of circuit fabrications and measurements in the experiment can be found in Note S9.

Figure 3c shows the variations of measured voltage with frequency. Three solid lines represent the voltage distributions when the perturbation capacitors are taken as ${{C}_\alpha }$= 0.1, 0.01 and 0.001 nF, respectively. It can be clearly seen that the splitting of the resonance frequency increases with the increase in perturbation, which corresponds to the simulated results in Fig. 2b. In Fig. 3d, we provide the sensitivity results based on the resonance frequency. The dots represent the experimental results, while two solid lines represent the theoretical sensitivities of third- and sixth-order RECs. Agreement between the experimental and theoretical results is observed again. A slight difference between the experimental results and the simulated results is caused by the loss and error of components in the circuit. The measured voltage distributions and sensitivities for the second-, fourth- and fifth-order RECs are given in Note S10, and are also consistent with the simulated results.

In the discussion above, by varying the capacitor ${{C}_\alpha }$, the ultra-sensitivity of the REC is shown. In fact, such sensors can have the good performance in the measurements of some realistic quantities, such as displacement, rotation-angle and liquid-level. In the following, we describe applications of the enhanced detection of tiny displacements. A displacement capacitor is fabricated as shown in Fig. 3b, corresponding to the theoretically designed scheme depicted in the dashed box at the bottom of Fig. 2a. It contains two ${\mathrm{75}} \times 20{\mathrm{0}}$ mm electrode plates (Electrode 1 and 2), as well as a displacement stage that can achieve the transverse and vertical motion of electrodes. In the experiment, Electrode 2 is kept unmoved, while the transverse and vertical motion of Electrode1 is controlled by adjusting the knob. In this way, we can adjust the overlapping area and spacing of two electrodes to achieve different capacitor values. The method for fabricating the displacement capacitor is given in Note S11. The relationship between displacements and capacitor values for this displacement capacitor is measured and presented in Fig. 3e. It is found that the variation of the value for the measured capacitor is linear with the change of displacement. When the displacement changes from 0 to 150 mm, the capacitor value changes from 1 to 200 *pF* accordingly. Considering that our REC can detect a minimum change of 1 *pF* in the capacitor, we can accurately read the corresponding variation of 0.75 mm in displacement. In our experiment, we use wires to connect the displacement capacitor to the first and last nodes in the circuit, replacing the capacitor ${{C}_\alpha }$ (the circle at the bottom of Fig. 3a) in Fig. 3a. By controlling the transverse motion of Electrode1, we successfully measure the sensitivity based on the displacement, as shown in Fig. 3f. The dots represent the experimental results, and two solid lines represent the theoretical results of the third- and sixth-order systems, respectively. We find that the experimental results based on the displacement are in accordance with the theoretical results. The inconsistencies between the dot and the line are 2-fold: one is from the minor mismatched relation between displacement and capacitance values, and the other is due to the loss of the electric components. In general, the high agreement between our experimental and theoretical results indicates that the ultra-sensitive detection of displacement is successfully realized in our designed RECs.

Previous studies have pointed out that the influence of noises on the designed sensors imposes a fundamental limit on sensitivity. In this case, the influence of noises should be clarified in our designed circuit sensors. In the electronic circuit, the main noise contributions are from shot noise, flicker noise and thermal noise [20,29,49,50]. Since shot noise mainly exists in circuits with tunneling diodes or vacuum tubes, and flicker noise can be significantly reduced below the level of thermal noise by choosing appropriate resistors, we aim for analyzing thermal noise in detail. Through experimental measurements, we find that the SNR of the circuit we designed is very stable and does not increase with an increase in order. This fully demonstrates the stability of our circuit. Detailed SNR analysis is given in Note S12.

## DISCUSSION AND CONCLUSION

The ultra-sensitive circuit sensors designed and fabricated above are based on printed circuit board (PCB) platforms. In fact, the corresponding on-chip ultra-sensitive sensing systems can also be realized by using a Complementary Metal Oxide Semiconductor (CMOS) process technology similar to those in ref. [50]. Three main advantages of the chip-implemented circuit sensor are described below. The first advantage is the easy configuration of components. Based on such a property, a more steady and reliable response for the output impedance is achieved through elaborately choosing the circuit components. The second advantage is the enormous reduction of the parasitic effect within the chip. Generally speaking, in the conventional PCB platform, the magnitudes of this parasitic capacitance and the fore-end capacitance are similar, which hinders the detection of ultra-small value capacitance. When referring to the chip, the parasitic capacitance can be nearly negligible, which greatly improves the capability to detect weak physical quantities. The third advantage is the increased operating frequency of the topological mode on the chip platform. Due to the very small value capacitances and inductances, such operating frequency of the chip can be three orders of magnitude higher than that of the PCB platform. Since the circuit components of the chip can be formulated exactly, the noise generated on the impedance spectrum is easily forecasted. Considering the three main advantages above, our design has the potential to be implemented as a novel ultra-sensitive sensor on a chip.

In conclusion, we have provided a new method for achieving ultra-sensitivity by reconstructing exceptional systems. Some eigenstates near the previous EPs have been used and non-reciprocal long-range couplings have been introduced in the reconstruction process, which results in the sensitivities of the reconstructed systems improving by several orders of magnitude compared to those based on EPs. Furthermore, we have designed and fabricated corresponding integrated circuits to demonstrate the scheme. It is noted that long-range couplings bring about enhanced sensing performance. As a comparison, the sensing effect will be degraded if the long-range couplings are removed. Details are shown in Note S13. Moreover, our proposed scheme demonstrates robustness to disorders. Even when large disorders are added into the proposed system, the ultra-sensitivity is still remarkable, which indicates that our design can be feasibly applied to other experimental platforms also. A full analysis of robustness in our design has been presented in Note S14. Our designed ultra-sensitive sensors are expected to find potential application in various fields [51] and provide a promising prospect for next-generation sensing techniques and devices.

## METHODS

### The realization of reconstructed exceptional systems

The eigenstates of the system *H* near the EP are applied to design the RES. In the construction, we choose the form of ${{H}_{\mathrm{1}}}$ as Equation (2) for convenience. This term illustrates the deviation from the EP Hamiltonian, and can be implemented easily. The Hamiltonian ${{H}_{\mathrm{1}}}$ plays the role of connecting between the first and last lattices, and adding the on-site potential on the first and last lattices only.


(2) \begin{eqnarray*}
{{H}_{\mathrm{1}}}{\mathrm{ \,=\, }}{{\left( {\begin{array}{@{}*{5}{c}@{}} {\mathrm{1}}&{\mathrm{0}}& \cdots &{\mathrm{0}}&{\mathrm{1}}\\ {\mathrm{0}}&{\mathrm{0}}& \cdots &{\mathrm{0}}&{\mathrm{0}}\\ \vdots & \vdots & \ddots & \vdots & \vdots \\ {\mathrm{0}}&{\mathrm{0}}& \cdots &{\mathrm{0}}&{\mathrm{0}}\\ {\mathrm{1}}&{\mathrm{0}}& \cdots &{\mathrm{0}}&{\mathrm{1}} \end{array}} \right)}_{N \times N}}.\end{eqnarray*}

Considering the Hamiltonian $H{\mathrm{ \,=\, }}{{H}_0} + \chi {{H}_1}$, it is found that ${{\varepsilon }_{\mathrm{1}}} = \frac{{\langle {{{\phi }_0}} |{{H}_1}| {{{\psi }_{\mathrm{0}}}} \rangle }}{{{{\varepsilon }_1}^{N - 1}}}{\mathrm{ \,=\, }}\frac{{\mathrm{1}}}{{{{\varepsilon }_1}^{N - 1}}}$, the other eigenvalues are ${{\varepsilon }_m} = {\mathrm{0}}( {2 \le m \le N} )$ and the states are $| {{{\psi }_{\mathrm{1}}}} \rangle {\mathrm{ \,=\, }}{{\varepsilon }_{\mathrm{1}}}| {{{\psi }_{{\mathrm{0,1}}}}} \rangle $, $| {{{\psi }_{\mathrm{2}}}} \rangle {\mathrm{ \,=\, }}{{\varepsilon }_{\mathrm{1}}}^{\mathrm{2}}| {{{\psi }_{{\mathrm{0,2}}}}} \rangle + {{\varepsilon }_2}| {{{\psi }_{{\mathrm{0,1}}}}} \rangle $ and $| {{{\psi }_{\mathrm{3}}}} \rangle {\mathrm{ \,=\, }}{{\varepsilon }_{\mathrm{1}}}^{\mathrm{3}}| {{{\psi }_{{\mathrm{0,3}}}}} \rangle + 2{{\varepsilon }_{\mathrm{1}}}{{\varepsilon }_2}| {{{\psi }_{{\mathrm{0,2}}}}} \rangle + {{\varepsilon }_3}| {{{\psi }_{{\mathrm{0,1}}}}} \rangle $. The detailed expression for ${{\varepsilon }_m}( {1 \le m \le N} )$, ${{\psi }_m}$ (${\mathrm{1}} \le m < N$) and derivation of states can be found in Note S1. We choose ${{\varepsilon }_{\mathrm{1}}} = 1$, the expressions for $| {{{\psi }_i}} \rangle = {{( {0,...,0,1,0,...,0} )}^T}$ where only the $( {i + 1} )$th element is non-zero. In this way the eigenstate becomes $| \psi \rangle {\mathrm{ \,=\, }}| {{{\psi }_{\mathrm{0}}}} \rangle {\mathrm{ + }}{{\chi }^{\frac{{\mathrm{1}}}{N}}}| {{{\psi }_1}} \rangle {\mathrm{ + }}{{\chi }^{\frac{{\mathrm{2}}}{N}}}| {{{\psi }_{\mathrm{2}}}} \rangle {\mathrm{ + }} \cdots + {{\chi }^{\frac{{N - 1}}{N}}}| {{{\psi }_{N - 1}}} \rangle = {{( {1,{{\chi }^{\frac{{\mathrm{1}}}{N}}},{{\chi }^{\frac{2}{N}}}, \cdots ,{{\chi }^{\frac{{N - 1}}{N}}}} )}^T}$. Following a similar procedure, the eigenstate of ${{H}^T}$ (${{H}^T}| \phi \rangle = {{\varepsilon }^T}| \phi \rangle $) is obtained as $| \phi \rangle {\mathrm{ \,=\, }}| {{{\phi }_{\mathrm{0}}}} \rangle {\mathrm{ + }}{{\chi }^{\frac{{\mathrm{1}}}{N}}}{{| \phi \rangle }_1}{\mathrm{ + }}{{\chi }^{\frac{{\mathrm{2}}}{N}}}| {{{\phi }_{\mathrm{2}}}} \rangle {\mathrm{ + }} \cdots {\mathrm{ + }}{{\chi }^{\frac{{N - 1}}{N}}}| {{{\phi }_3}} \rangle = {{( {{{\chi }^{\frac{{N - 1}}{N}}}, \cdots ,{{\chi }^{\frac{{\mathrm{2}}}{N}}},{{\chi }^{\frac{{\mathrm{1}}}{N}}},1} )}^T}$. The expression of the state is $| {{{\phi }_i}} \rangle = {{( {0,...,0,1,0,...,0} )}^T}$, where only the $( {N - i} )$th element is non-zero. Therefore, the Hamiltonian ${{H}_{{\mathrm{RES}},N}}{\mathrm{ \,=\, }}{{\chi }^{\frac{N}{{N - 1}}}}| \psi \rangle \langle \phi |$ for the RES is obtained as


(3) \begin{eqnarray*}
{{H}_{{\mathrm{RES}},N}} = \left( {\begin{array}{@{}*{4}{c}@{}} {\mathrm{1}}&{{{\chi }^{ - \frac{{\mathrm{1}}}{N}}}}& \cdots &{{{\chi }^{ - \frac{{N - 1}}{N}}}}\\ {{{\chi }^{\frac{{\mathrm{1}}}{N}}}}&1& \cdots &{{{\chi }^{ - \frac{{N - 2}}{N}}}}\\ \vdots & \vdots & \ddots & \vdots \\ {{{\chi }^{\frac{{N - 1}}{N}}}}& \cdots &{{{\chi }^{\frac{{\mathrm{1}}}{N}}}}&1 \end{array}} \right).\end{eqnarray*}

For example, when $N = 2 {{H}_{{\mathrm{RES}},2}} = $  
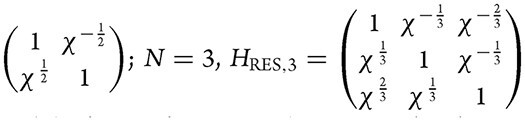
; and the forms of ${{H}_{{\mathrm{RES}},N}}$ with any *N* can be obtained as discussed above.

### The sensing in reconstructed exceptional systems

In our study, the sensed term displays the connection between the first and last lattices. When the perturbation strength induced by the sensed item is $\alpha $, the Hamiltonian for the sensed term is 
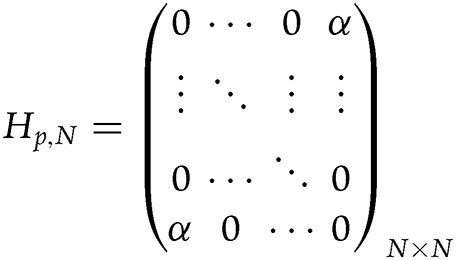
.

When the order is chosen as $N = 2$, the Hamiltonian ${{H}_{{\mathrm{RES}},2}}$ for the RES is ${{H}_{{\mathrm{RES}},2}} = \left ( { {_{X^\frac{1}{2}}^{1}}} \ { {_{1}^{X^-\frac{1}{2}}}}\right )$. When applying this system to sense the perturbation ${{H}_{p,2}}$, the Hamiltonian for the total system is ${{H}_{{\mathrm{RES}},2}} + H_{p,2}= \left ( { {_{X {^\frac{1}{2}} + \alpha}^{1}}} \ { {_{1}^{X ^-\frac{1}{2}+\alpha}}}\right )$. The difference of eigenvalues is $\Delta {{E}_{{\mathrm{RES}},2}} = 2\sqrt {1 + \alpha {{\chi }^{\frac{1}{2}}} + \alpha {{\chi }^{ - \frac{1}{2}}} + {{\alpha }^2}} $ and the sensitivity with respect to the perturbation strength $\alpha $ is $\frac{{\partial \Delta {{E}_{{\mathrm{RES}},2}}}}{{\partial \alpha }} = \frac{{( {{\mathrm{1}} + \chi } ) + 2\sqrt \chi \alpha }}{{\sqrt {\chi + {{\chi }^{\frac{1}{2}}}( {{\mathrm{1}} + \chi } )\alpha + \chi {{\alpha }^2}} }}$. In our study, it is assumed that the perturbation strength $\alpha$ is rather small which satisfies $\alpha < {\mathrm{1}}$, $\chi < {\mathrm{1}}$ and $\sqrt \chi < \alpha $, we can have $\frac{{\partial \Delta {{E}_{{\mathrm{RES}},2}}}}{{\partial \alpha }} \approx \frac{{\mathrm{1}}}{{{{\chi }^{\frac{{\mathrm{1}}}{{\mathrm{4}}}}}{{\alpha }^{\frac{{\mathrm{1}}}{{\mathrm{2}}}}}}}$. As a comparison, we use the non-Hermitian system possessing the second-order EP to sense the perturbation. The Hamiltonian possessing the second-order EP is ${{H}_{\mathrm{0}}} {\rm = } \left ( { {_{0}^{0}}} \ { {_{0}^{1}}}\right )$, and the Hamiltonian for the total system is ${{H}_{\mathrm{0}}} + {{H}_{p,2}} = \left ( { {_{\alpha}^{0}}} \ { {_{0}^{1+\alpha}}}\right )$. The difference of eigenvalues is $\Delta {{E}_{{\mathrm{EP}},2}} = 2\sqrt {\alpha ( {1 + \alpha } )} $ and the sensitivity with respect to perturbation strength $\alpha $ is $\frac{{\partial \Delta {{E}_{{\mathrm{EP}},2}}}}{{\partial \alpha }} = \frac{{{\mathrm{1}} + 2\alpha }}{{\sqrt {\alpha ( {{\mathrm{1}} + \alpha } )} }}$. When the perturbation strength is rather small $\alpha < {\mathrm{1}}$, the sensitivity is simplified as $\frac{{\partial \Delta {{E}_{{\mathrm{EP}},2}}}}{{\partial \alpha }} \approx \frac{{\mathrm{1}}}{{\sqrt \alpha }}$.

Moreover, for any order *N*, we apply the Hamiltonian ${{H}_{{\mathrm{RES}},N}}$ to sense the perturbation ${{H}_{p,N}}$. In our study, it is assumed that when the perturbation strength $\alpha $ is rather small, the sensitivity can be expressed as $\frac{{\partial \Delta {{E}_{{\mathrm{RES}},N}}}}{{\partial \alpha }} \approx \frac{{\mathrm{1}}}{{{{\chi }^{\frac{{N - 1}}{{{\mathrm{2}}N}}}}{{\alpha }^{\frac{{\mathrm{1}}}{{\mathrm{2}}}}}}}$.

## Supplementary Material

nwae278_Supplemental_File
