# Supplementary material for: Ultra-sensitivity in reconstructed exceptional systems
Source: Natl Sci Rev. 2024 Aug 16;11(12):nwae278. doi: 10.1093/nsr/nwae278 (PMC11562849; doi:10.1093/nsr/nwae278)
Supplement: nwae278_Supplemental_File [file nwae278_supplemental_file.pdf]

Supplementary Information for

"Ultra-sensitivity in reconstructed exceptional systems"

Tian Chen[1*], Deyuan Zou[1*], Zilong Zhou[2], Ruiguo Wang[2], Yue Feng[2+], Houjun Sun[3#], and Xiangdong Zhang[1$]

[1]*Key Laboratory of advanced optoelectronic quantum architecture and measurements of Ministry of Education, Beijing Key Laboratory of Nanophotonics & Ultrafine Optoelectronic Systems, School of Physics, Beijing Institute of Technology, 100081, Beijing, China*

[2]*School of Mechatronical Engineering, Beijing Institute of Technology, Beijing 100081, China*

[3]*Beijing Key Laboratory of Millimeter wave and Terahertz Techniques, School of Information and Electronics, Beijing Institute of Technology, Beijing 100081, China*

**These authors contributed equally to this work.* [$#+]*Author to whom any correspondence should be addressed. E-mail: zhangxd@bit.edu.cn; sunhoujun@bit.edu.cn; fengyue@bit.edu.cn*

Supplementary Note 1. The eigenstates and eigenvalues for the non-Hermitian Hamiltonian near the EP.

Supplementary Note 2. The sensitivity for the RES with N>6.

Supplementary Note 3. The implementation of non-reciprocal capacitors.

Supplementary Note 4. The groundings of circuits.

Supplementary Note 5. The correspondence between the circuit Laplacian and lattice Hamiltonian.

Supplementary Note 6. Theoretical calculation of circuit sensitivity.

Supplementary Note 7. The theoretical derivation of resonance frequencies for third- and sixth-order RECs.

Supplementary Note 8. The sensitivity of the second-, fourth-and fifth-order RECs, and the comparison of sensitivity between the RECs and EP circuits.

Supplementary Note 9. Circuit fabrications and measurements in experiment.

Supplementary Note 10. Experimental voltage distribution voltage distributions and sensitivities for the other orders.

Supplementary Note 11. The fabrication of displacement capacitor.

Supplementary Note 12. The analysis of signal-to-noise ratio in the reconstructed exponential circuit.

Supplementary Note 13. The effect of long-range couplings in the sensing.

Supplementary Note 14. The robustness of the REC.

Supplementary Note 1. The eigenstates and eigenvalues for the non-Hermitian Hamiltonian near the EP.

In this section, the eigenstates and eigenvalues for the non-Hermitian Hamiltonian near the EP are provided. We start from the non-Hermitian Hamiltonian H_0 possessing the *N*th-order EP. The H_0 can be expressed as

$$H_0=\begin{pmatrix} 0 & 1 & 0 & \cdots & 0 \\ 0 & 0 & 1 & 0 & \vdots \\ 0 & \cdots & 0 & \ddots & 0 \\ \vdots & 0 & \cdots & 0 & 1 \\ 0 & \cdots & 0 & \cdots & 0 \end{pmatrix}_{N\times N} . \quad \text{(S1)}$$

When solving the eigen-equation for H_0, due to the *N*th-order EP, not only *N* eigenvalues but also the corresponding *N* states coalesce. Therefore, a linear-independent eigenstate can be obtained as $H_0|\psi_0\rangle=\varepsilon_0|\psi_0\rangle$, and there exist $N-1$ associated eigenstates that form the so-called Jordan chain. A group of Jordan chain is $|\psi_0\rangle$, $|\psi_{0,1}\rangle$, $|\psi_{0,2}\rangle$, …, $|\psi_{0,N\text{-}1}\rangle$, which satisfy *N* equations as $H_0|\psi_0\rangle=\varepsilon_0|\psi_0\rangle$, $H_0|\psi_{0,1}\rangle=\varepsilon_0|\psi_{0,1}\rangle+|\psi_0\rangle$, $H_0|\psi_{0,2}\rangle=\varepsilon_0|\psi_{0,2}\rangle+|\psi_{0,1}\rangle$,…, $H_0|\psi_{0,N\text{-}1}\rangle=\varepsilon_0|\psi_{0,N\text{-}1}\rangle+|\psi_{0,N\text{-}2}\rangle$.

Another group of Jordan chain $|\phi_0\rangle$, $|\phi_{0,1}\rangle$, $|\phi_{0,2}\rangle$, …, $|\phi_{0,N\text{-}1}\rangle$, can be obtained from $H_0{}^T$ as $H_0{}^T|\phi_0\rangle=\varepsilon_0|\phi_0\rangle$, $H_0{}^T|\phi_{0,1}\rangle=\varepsilon_0|\phi_{0,1}\rangle+|\phi_0\rangle$, $H_0{}^T|\phi_{0,2}\rangle=\varepsilon_0|\phi_{0,2}\rangle+|\phi_{0,1}\rangle$,…, $H_0{}^T|\phi_{0,N\text{-}1}\rangle=\varepsilon_0|\phi_{0,N\text{-}1}\rangle+|\phi_{0,N\text{-}2}\rangle$, where the superscript "*T*" denotes the transpose operation. Combining two Jordan chains above, we have

$$(H_0-\varepsilon_0 I)|\psi_{0,i}\rangle=|\psi_{0,i-1}\rangle,$$

$$\langle\phi_{0,i}|(H_0-\varepsilon_0 I)=\langle\phi_{0,i-1}|,\quad (i\geq 1, |\psi_{0,0}\rangle\ (|\phi_{0,0}\rangle) \text{ is just } |\psi_0\rangle\ (|\phi_0\rangle));$$

$$(H_0-\varepsilon_0 I)|\psi_0\rangle=0,\ \langle\phi_0|(H_0-\varepsilon_0 I)=0. \quad \text{(S2)}$$

Considering the expression of H_0, the eigenvalue ε_0 is zero, and the corresponding

eigenstate is $|\psi_0\rangle=(1,0,...,0)^T$. Therefore, the expressions for the Jordan chain are $|\psi_{0,i}\rangle=(0,...,0,1,0,...,0)^T$ where only the $(i+1)$th element is non-zero and the other elements are zero. The left eigenstate $|\phi_0\rangle=(0,...,0,1)^T$, and the expressions for the Jordan chain are $|\phi_{0,i}\rangle=(0,...,0,1,0,...,0)^T$ where only the $(N-i)$th element is non-zero and the other elements are also zero. These two groups of Jordan chains have the orthogonal relations, $\langle\phi_0|\psi_0\rangle=0$, $\langle\phi_{0,1}|\psi_0\rangle=\langle\phi_0|\psi_{0,1}\rangle$, $\langle\phi_{0,N\text{-}2}|\psi_0\rangle=\langle\phi_{0,N\text{-}3}|\psi_{0,1}\rangle=\cdots=\langle\phi_0|\psi_{0,N\text{-}2}\rangle=0$, $\langle\phi_{0,N\text{-}1}|\psi_0\rangle=\langle\phi_{0,N\text{-}2}|\psi_{0,1}\rangle=\cdots=\langle\phi_0|\psi_{0,N\text{-}1}\rangle\neq 0$.

Then, we move to the case where the parameters of system are near the EP. In this case, the Hamiltonian of the system is H . The H can be expanded around the *N*th-order EP Hamiltonian H_0 as $H=H_0+\chi H_1$. The parameter χ evaluates the deviation from the *N*th-order EP. The eigen-equation is $H|\psi\rangle=\varepsilon|\psi\rangle$. We assume that the eigenvalue ε and the corresponding eigenstate $|\psi\rangle$ can also be expanded in the orders of χ , $\varepsilon=\varepsilon_0+\chi^{\frac{1}{N}}\varepsilon_1+\chi^{\frac{2}{N}}\varepsilon_2+\cdots+\chi^{\frac{n}{N}}\varepsilon_n$ and $|\psi\rangle=|\psi_0\rangle+\chi^{\frac{1}{N}}|\psi_1\rangle+\chi^{\frac{2}{N}}|\psi_2\rangle+\cdots+\chi^{\frac{n}{N}}|\psi_n\rangle$, where we have $H_0|\psi_0\rangle=\varepsilon_0|\psi_0\rangle$. When putting the eigenvalue ε and the corresponding eigenstate $|\psi\rangle$ into the eigen-equation $H|\psi\rangle=\varepsilon|\psi\rangle$, we obtain a series of equations in the order of $\chi^{\frac{1}{N}}$, $\chi^{\frac{2}{N}}$ and so on.

$$\chi^{\frac{1}{N}}:\ H_0|\psi_1\rangle=\varepsilon_0|\psi_1\rangle+\varepsilon_1|\psi_0\rangle$$

$$\chi^{\frac{2}{N}}:\ H_0|\psi_2\rangle=\varepsilon_0|\psi_2\rangle+\varepsilon_1|\psi_1\rangle+\varepsilon_2|\psi_0\rangle$$

$$\cdots$$

$$\chi^{\frac{N-1}{N}}:\ H_0|\psi_2\rangle=\varepsilon_0|\psi_{N\text{-}1}\rangle+\varepsilon_1|\psi_{N\text{-}2}\rangle+\cdots+\varepsilon_{N\text{-}1}|\psi_0\rangle$$

$$\chi^{\frac{N}{N}}:\ H_0|\psi_N\rangle+H_1|\psi_0\rangle=\varepsilon_0|\psi_N\rangle+\varepsilon_1|\psi_{N-1}\rangle+\cdots+\varepsilon_N|\psi_0\rangle$$

$$\chi^{\frac{N+1}{N}}:\ H_0|\psi_{N+1}\rangle+H_1|\psi_1\rangle=\varepsilon_0|\psi_{N+1}\rangle+\varepsilon_1|\psi_N\rangle+\cdots+\varepsilon_{N+1}|\psi_0\rangle$$

$$\cdots$$

$$\chi^{\frac{N+j-1}{N}}: \ H_0|\psi_{N+j-1}\rangle + H_1|\psi_{j-1}\rangle = \varepsilon_0|\psi_{N+j-1}\rangle + \varepsilon_1|\psi_{N+j-2}\rangle + \cdots + \varepsilon_{N+j-1}|\psi_0\rangle$$

$$\cdots \tag{S3}$$

When comparing Eq. (S2) with Eq. (S3), we have $(H_0-\varepsilon_0)|\psi_1\rangle = \varepsilon_1|\psi_0\rangle$ and $|\psi_1\rangle = \varepsilon_1|\psi_{0,1}\rangle$; $(H_0-\varepsilon_0)|\psi_2\rangle = \varepsilon_1|\psi_1\rangle + \varepsilon_2|\psi_0\rangle$ and $|\psi_2\rangle = \varepsilon_1^2|\psi_{0,2}\rangle + \varepsilon_2|\psi_{0,1}\rangle$. And following this procedure, we can have the expressions $|\psi_m\rangle$ ($1 \le m < N$) with the combinations of $|\psi_{0,s}\rangle$ ($1 \le s < N$). And the expression of ε_m can be obtained by left-multiplied $\langle\phi_0|$ as,

$$\varepsilon_m = \frac{\langle\phi_0|H_1|\psi_{m-1}\rangle - \langle\phi_0|\left(\varepsilon_1|\psi_{N+m-2}\rangle + \varepsilon_2|\psi_{N+m-3}\rangle + \cdots + \varepsilon_{m-1}|\psi_N\rangle\right)}{\varepsilon_1^{N-1}}. \tag{S4}$$

Therefore, the expression of ε_m can be shown with $\varepsilon_{m'}(1 \le m' < m)$ and $\langle\phi_0|\psi_{N-1+m'}\rangle$. In this way, we can obtain the expression for the eigenvalue ε and the corresponding eigenstate $|\psi\rangle$ in any orders of χ.

Supplementary Note 2. The sensitivity for the RES with *N*>6

In this section, the sensitivity for the higher-order (*N*>6) RES is provided. In the main text, we provide the sensitivity for the RES with the order *N* from 2 to 6. It is obviously seen that, with the increase of order *N*, the value of sensitivity also increases. Here, the sensitivity for the higher-order (*N*>6) RES is provided, and it is better than that for the sixth-order RES.

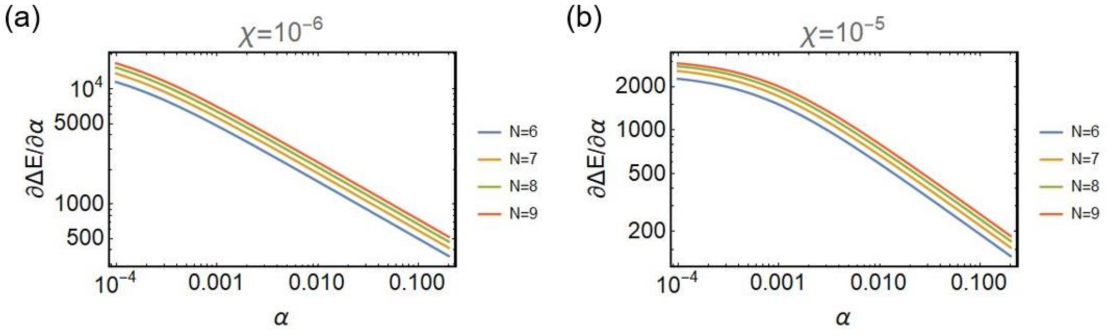

Supporting Figure 1. The sensitivity for the high-order RES. In (a) and (b), the sensitivity $\frac{\partial \Delta E_N}{\partial \alpha}$ with the perturbation strength α . The blue, yellow, green, and red solid lines,

correspond to the reconstructed exceptional system with *N* from 6 to 9. (a) $\chi = 10^{-6}$ and (b) $\chi = 10^{-5}$.

In Supporting Figure 1, we have chosen the order from 6 to 9, which are shown as blue, yellow, green, and red solid lines. The parameter χ to construct the RES is chosen as $\chi = 10^{-6}$ in Supporting Figure 1(a) and $\chi = 10^{-5}$ in Supporting Figure 1(b), respectively. It is found that, in the range of the perturbation strength $\alpha \in [0.0001, 0.1]$, the higher order leads to the better sensitivity $\frac{\partial \Delta E_N}{\partial \alpha}$. Moreover, as demonstrated in Section 2 of Method, for any order N, we apply the Hamiltonian $H_{\mathrm{RES},N}$ to sense the perturbation $H_{p,N}$. In our study, it is assumed that the perturbation strength is rather small, we can have the sensitivity with respect to perturbation strength α as shown by the theoretic derivation, $\frac{\partial \Delta E_{\mathrm{RES},N}}{\partial \alpha} \approx \frac{1}{\chi^{\frac{N-1}{2N}} \alpha^{\frac{1}{2}}}$.

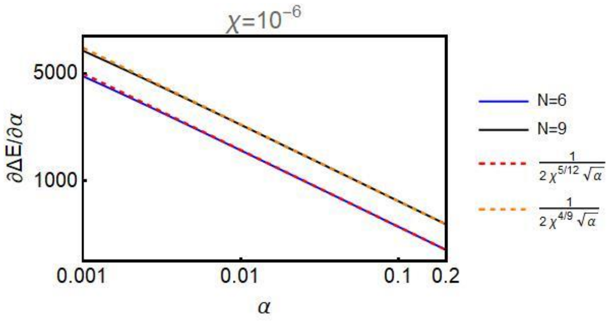

Supporting Figure 2. The sensitivity for the sixth and ninth-order RESs. The blue and black solid lines are numerical results of sensitivity for *N*=6 and *N*=9, respectively. The red and yellow dashed lines represent the results from the theoretic derivation above. The parameter $\chi = 10^{-6}$.

In Supporting Figure 2, we have chosen the sixth and ninth-order RESs to show the sensitivity. The numerical results are shown as blue and black solid lines for the sixth and ninth-order RESs, respectively. The parameter χ to construct the RES is chosen as $\chi = 10^{-6}$. We also provide the theoretic derivation $\frac{\partial \Delta E_{\mathrm{RES},N}}{\partial \alpha} \approx \frac{1}{\chi^{\frac{N-1}{2N}} \alpha^{\frac{1}{2}}}$ for different orders. When *N*=6, this

equation is approximately as $\frac{1}{\chi^{\frac{5}{12}}\alpha^{\frac{1}{2}}}$; and when N=9, this equation is approximately as $\frac{1}{\chi^{\frac{4}{9}}\alpha^{\frac{1}{2}}}$. These theoretic derivation results are shown as red and yellow dashed lines, respectively. It is clearly found that, such theoretic derivation agrees with the numerical results well. Moreover, the limit of sensitivity with N is approximately as $\frac{\partial \Delta E_{\mathrm{RES},N}}{\partial \alpha} \approx \frac{1}{\chi^{\frac{N-1}{2N}}\alpha^{\frac{1}{2}}} \approx \frac{1}{\chi^{\frac{1}{2}}\alpha^{\frac{1}{2}}}(N \to \infty)$.

Supplementary Note 3. The implementation of non-reciprocal capacitors.

In this section, we clarify the implementation of the non-reciprocal capacitor. In Supporting Figure 3, we show the details of the non-reciprocal capacitor. The blue dotted rectangle represents the voltage follower, which consists of an ideal operational amplifier. By connecting capacitor $2C_i$ in series with the voltage follower, and the other capacitance $(C_{i-1} - C_i)$ in parallel is added. In this case, the non-reciprocal capacitance with the value being $C_{i-1} \pm C_i$ is achieved. Based on the characteristic that voltage follower can block the input current while keeping the output voltage stable, we get the following equation by carrying out Kirchhoff's law on the circuit node 1 and node 2

$$I_{i-1} = i\omega(C_{i-1} - C_i)(V_{i-1} - V_i),$$

$$I_i = i\omega(C_{i-1} - C_i)(V_i - V_{i-1}) + 2i\omega C_i(V_i - V_{i-1}). \tag{S5}$$

The Eq. (S5) can be re-expressed in a matrix form as:

$$\begin{pmatrix} I_{i-1} \\ I_i \end{pmatrix} = J\begin{pmatrix} V_{i-1} \\ V_i \end{pmatrix} = i\omega\begin{bmatrix} -(C_{i-1} - C_i) & (C_{i-1} - C_i) \\ (C_{i-1} + C_i) & -(C_{i-1} + C_i) \end{bmatrix}\begin{pmatrix} V_{i-1} \\ V_i \end{pmatrix}, \tag{S6}$$

where ***J*** is the admittance matrix of the circuit structure. It is seen that the conductance matrix is a non-Hermitian matrix, and two off-diagonal elements of $C_{i-1} \pm C_i$ represent effective values of the connecting capacitor between two nodes.

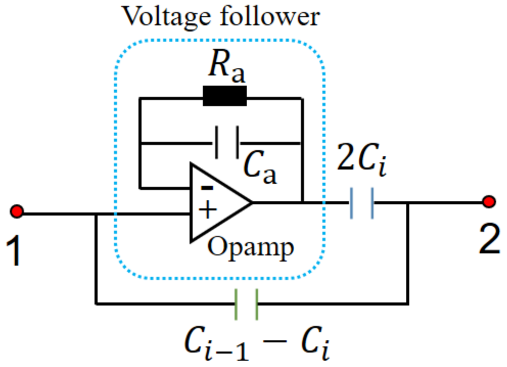

Supporting Figure 3. The model of the non-reciprocal capacitor.

Supplementary Note 4. The groundings of circuits.

To obtain the circuit Laplacian J, proper grounding elements should be connected on each node. However, in our circuit, the groundings of each node vary with the change of N. The principle of groundings remains unchanged, and the on-site potential of each node is the same. For example, in our circuit with N nodes, each node can be connected to a maximum of two $C_1 - C_2$ couplings and one $2C_2$ coupling. Therefore, we made the on-site potential of each node for C_1 and C_2 be $2(C_1 - C_2) + 2C_2 = 2C_1$. When each node is connected to a maximum of one $C_{N-1} - C_N$ and $2C_N$ couplings, we made the on-site potential of each node for C_{N-1} and C_N be $C_{N-1} - C_N + 2C_N = C_{N-1} + C_N$. Supporting Figure 4(a) shows the detailed groundings of our circuit with N nodes. Especially, we give the details of groundings for third- and sixth-order reconstructed exceptional systems in Supporting Figure 4(b) and 4(c), respectively.

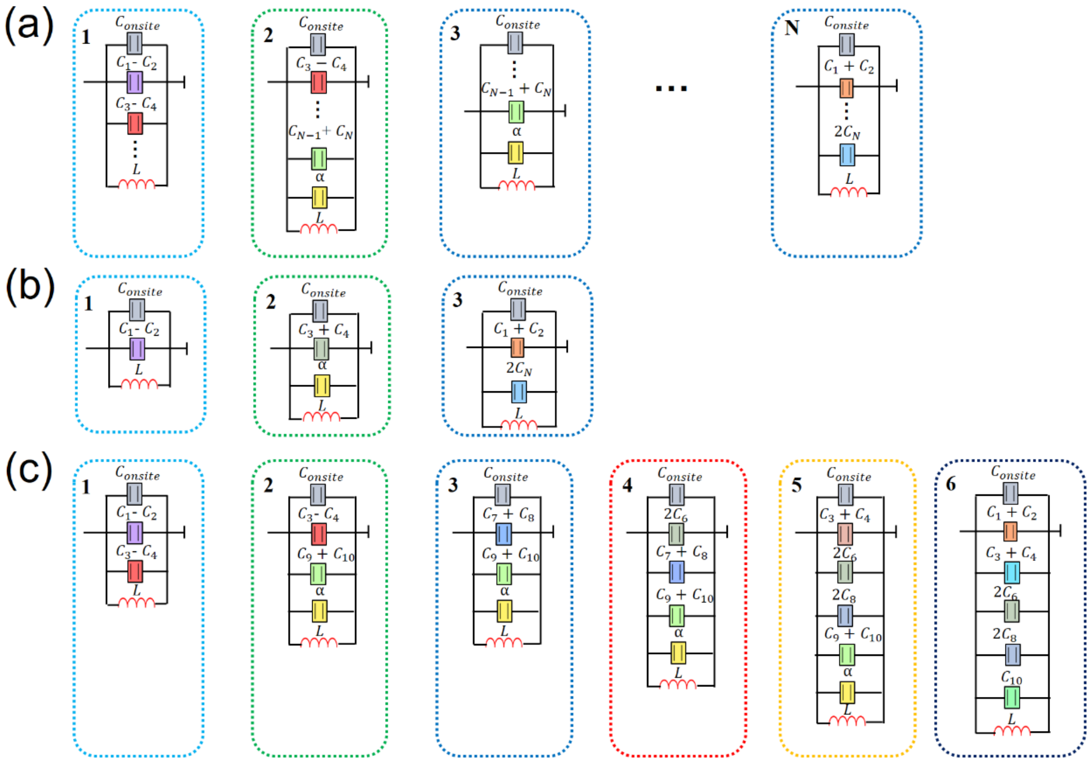

Supporting Figure 4. The groundings in our reconstructed exceptional systems. (a) The groundings of our circuit with N nodes. (b) The groundings for the third-order reconstructed exceptional system. (c) The groundings for the sixth-order reconstructed exceptional system.

Supplementary Note 5. The correspondence between the circuit Laplacian and lattice Hamiltonian.

In this section, the correspondence between the eigen-equation between the lattice model and our designed circuit is presented. Based on the Kirchhoff's law, the voltage and current at the node have the relation:

$$I_m = i\omega C_{inter} \sum_{\langle n \rangle} (V_n - V_m) - i\omega C_g V_m + \frac{iV_m}{\omega L_g}, \tag{S7}$$

where I_m and V_m are the net current and voltage of the node m with the angular frequency ω, L_g and C_g are the inductance and capacitance between the node m and ground, respectively. C_{inter} is the capacitance between the nodes m and n. In our study, the capacitance C_{inter} between different nodes m and n are $C_{i-1} \pm C_i$ ($i = 2, 4, \ldots, 2N-2$), which has been stated in the main text. $\langle n \rangle$ indicates the summation confined to other connected nodes. Therefore, the

currents flowing into each node can be written as

$$
\begin{aligned}
&I_1 = i\omega(C_1+C_2)(V_1-V_2)+i\omega(C_3+C_4)(V_1-V_3)+...+i\omega(C_{2N-3}+C_{2N-2}+C_\alpha)(V_1-V_N) \\
&+i\omega(C_1-C_2+C_3-C_4+...)V_1+i\omega(C_{onsite})V_1-\frac{iV_1}{\omega L}, \\
&I_2 = i\omega(C_1-C_2)(V_2-V_1)+i\omega(C_1+C_2)(V_2-V_3)+...+i\omega(C_{2N-5}+C_{2N-4})(V_2-V_N) \\
&+i\omega(C_3-C_4+...+C_{2N-3}+C_{2N-2}+C_\alpha)V_2+i\omega(C_{onsite})V_2-\frac{iV_2}{\omega L}, \\
&I_3 = i\omega(C_3-C_4)(V_3-V_1)+i\omega(C_1-C_2)(V_3-V_2)+...+i\omega(C_{2N-7}+C_{2N-6})(V_3-V_N) \\
&+i\omega(...C_{2N-3}+C_{2N-2}+C_\alpha)V_3+i\omega(C_{onsite})V_3-\frac{iV_3}{\omega L}, \\
&..... \\
&I_N = i\omega(C_{N-1}-C_N+C_\alpha)(V_{2N-3}-V_{2N-2})+i\omega(C_{2N-5}-C_{2N-4})(V_N-V_2)+...+i\omega(C_1-C_2)(V_N-V_{N-1}) \\
&+i\omega(C_1+C_2+...+2C_{2N-2})V_N+i\omega(C_{onsite})V_N-\frac{iV_N}{\omega L}.
\end{aligned}
\tag{S8}
$$

When it is assumed that there is no external source, the current flowing into the node is zero. In this case, Eq. (S8) becomes:

$$
\begin{aligned}
&\left[i\omega(2C_1+2C_3+...+C_{2N-3}+C_{2N-2}+C_\alpha)+i\omega C_{onsite}-\frac{i}{\omega L}\right]V_1 = i\omega(C_1+C_2)V_2+i\omega(C_3+C_4)V_3+...+i\omega(C_{2N-3}+C_{2N-2}+C_\alpha)V_N \\
&\left[i\omega(2C_1+2C_3+...+C_{2N-3}+C_{2N-2}+C_\alpha)+i\omega C_{onsite}-\frac{i}{\omega L}\right]V_2 = i\omega(C_1-C_2)V_1+i\omega(C_1+C_2)V_3+...+i\omega(C_{2N-5}+C_{2N-4})V_N \\
&\left[i\omega(2C_1+2C_3+...+C_{2N-3}+C_{2N-2}+C_\alpha)+i\omega C_{onsite}-\frac{i}{\omega L}\right]V_3 = i\omega(C_3-C_4)V_1+i\omega(C_1-C_2)V_2+...+i\omega(C_{2N-7}+C_{2N-6})V_N \\
&..... \\
&\left[i\omega(2C_1+2C_3+...+C_{2N-3}+C_{2N-2}+C_\alpha)+i\omega C_{onsite}-\frac{i}{\omega L}\right]V_N = i\omega(C_{2N-3}-C_{2N-2}+C_\alpha)V_1+i\omega(C_{2N-5}-C_{2N-4})V_2+...+i\omega(C_1-C_2)V_{N-1} \\
&.
\end{aligned}
\tag{S9}
$$

The Eq. (S9) above can be recast into a matrix form,

$$
\beta\begin{pmatrix} V_1 \\ ... \\ V_N \end{pmatrix} = i\omega\begin{bmatrix} -C_{onsite} & C_1+C_2 & C_3+C_4 & ... & C_{2N-3}+C_{2N-2}+C_\alpha \\ C_1-C_2 & -C_{onsite} & C_1+C_2 & ... & C_{2N-5}+C_{2N-4} \\ C_3-C_4 & C_1-C_2 & -C_{onsite} & ... & ... \\ ... & ... & ... & ... & C_1+C_2 \\ C_{2N-3}-C_{2N-2}+C_\alpha & C_{2N-5}-C_{2N-4} & ... & C_1+C_2 & -C_{onsite} \end{bmatrix}\begin{pmatrix} V_1 \\ ... \\ V_N \end{pmatrix},
\tag{S10}
$$

where $\beta = i\omega(2C_1+2C_3+...+C_{2N-3}+C_{2N-2}+C_\alpha)-\frac{i}{\omega L}$ and $\omega = 2\pi f$. The circuit Laplacian can be obtained as

$$J=\begin{bmatrix} -C_{onsite} & C_1+C_2 & C_3+C_4 & \cdots & C_{2N-3}+C_{2N-2}+C_\alpha \\ C_1-C_2 & -C_{onsite} & C_1+C_2 & \cdots & C_{2N-5}+C_{2N-4} \\ C_3-C_4 & C_1-C_2 & -C_{onsite} & \cdots & \cdots \\ \cdots & \cdots & \cdots & \cdots & C_1+C_2 \\ C_{2N-3}-C_{2N-2}+C_\alpha & C_{2N-5}-C_{2N-4} & \cdots & C_1+C_2 & -C_{onsite} \end{bmatrix}. \quad \text{(S11)}$$

If the parameters are chosen as $C_{onsite}=-1$, $C_1+C_2=\chi^{-1/N}$, $C_3+C_4=\chi^{-2/N}$,…, $C_{2N-3}+C_{2N-2}=\chi^{-(N-1)/N}$ and $C_1-C_2=\chi^{1/N}$, $C_3-C_4=\chi^{2/N}$,…, $C_{2N-3}+C_{2N-2}=\chi^{(N-1)/N}$, the circuit Laplacian has an excellent correspondence to the lattice model Hamiltonian in Eq. (1). Besides, the relation between the frequency f and eigenvalues E_{REC} is addressed below.

$$\beta = i\omega\left(2C_1+2C_3+\ldots+C_{2N-3}+C_{2N-2}+C_\alpha\right)-\frac{i}{\omega L}=E_{\mathrm{REC}}. \quad \text{(S12)}$$

Therefore, we can convert the resonance frequency for each C_α into the eigenvalue E_{REC} of the REC, which corresponds to the eigenvalue E_{RES} of the total Hamiltonian $H_{\mathrm{RES},N}+H_P$. Thus, the simulated results for the variation of the eigenvalue difference ΔE_{REC} with the difference of perturbations C_α can be obtained. Furthermore, based on the eigenvalue difference ΔE_{REC}, we can obtain the sensitivity $\frac{\partial \Delta E_{\mathrm{REC}}}{\partial C_\alpha}$ for our circuit.

Supplementary Note 6. Theoretical calculation of circuit sensitivity.

In this section, based on circuit Laplacian *J*, the derivation of sensitivity in our designed circuit is provided. The specific circuit Laplacian *J* is shown in Eq. (S11).

When the total number of nodes in the circuit is $N=2$, the Laplacian is $J_{N=2}=\begin{pmatrix} 1 & C_1+C_2+C_\alpha \\ C_1-C_2+C_\alpha & 1 \end{pmatrix}$. The difference of eigenvalues is $\Delta E_{\mathrm{REC}}=2\sqrt{1+C_\alpha\left(C_1-C_2\right)+C_\alpha\left(C_1+C_2\right)+C_\alpha{}^2}$ and the sensitivity with respect to the perturbation C_α is $\frac{\partial \Delta E_{\mathrm{REC}}}{\partial C_\alpha}=\frac{\left(1+\left(C_1-C_2\right)^2\right)+2\left(C_1-C_2\right)C_\alpha}{\sqrt{\left(C_1-C_2\right)^2+\left(C_1-C_2\right)\left(1+\left(C_1-C_2\right)^2\right)C_\alpha+\left(C_1-C_2\right)^2 C_\alpha{}^2}}$.

In our study, it is assumed that the perturbation is rather small, which is reflected as the value

of C_α<1, $(C_1-C_2)^2$<1, and $C_1-C_2<C_\alpha$, we can have $\frac{\partial \Delta E_{\mathrm{REC}}}{\partial C_\alpha}\approx\frac{1}{(C_1-C_2)^{\frac{1}{2}}C_\alpha^{\frac{1}{2}}}$.

When the total number of nodes in the circuit is $N=3$, the Laplacian is $J_{N=3}=\begin{pmatrix}1 & C_1+C_2 & C_3+C_4+C_\alpha\\ C_1-C_2 & 1 & C_1+C_2\\ C_3-C_4+C_\alpha & C_1-C_2 & 1\end{pmatrix}$. The expressions of eigenvalues are complicated, so we do not provide the detailed expressions here. In our study, it is also assumed that the perturbation is rather small, we can have the sensitivity with respect to the perturbation strength C_α is $\frac{\partial \Delta E_{\mathrm{REC}}}{\partial C_\alpha}\approx\frac{1}{(C_1-C_2)C_\alpha^{\frac{1}{2}}}$.

Similar to the above cases, when the total number of nodes in the circuit is $N=4$, we have the sensitivity with respect to the perturbation strength C_α is $\frac{\partial \Delta E_{\mathrm{REC}}}{\partial C_\alpha}\approx\frac{1}{(C_1-C_2)^{\frac{3}{2}}C_\alpha^{\frac{1}{2}}}$.

For any N, in our study, it is assumed that the perturbation strength is rather small, we have the difference of eigenvalues is $\Delta E_{\mathrm{REC}}\approx(C_1-C_2)^{-\left(\frac{N-1}{2}\right)}C_\alpha^{\frac{1}{2}}$ and the sensitivity with respect to the perturbation strength C_α is $\frac{\partial \Delta E_{\mathrm{REC}}}{\partial C_\alpha}\approx\frac{1}{(C_1-C_2)^{\frac{N-1}{2}}C_\alpha^{\frac{1}{2}}}$.

Supplementary Note 7. The theoretical derivation of resonance frequencies for third- and sixth-order RECs.

In this section, we will provide a detailed introduction to the selection of elements and theoretical derivation of resonance frequencies for the third- and sixth-order RECs. According to Eq. (S10) in Supplementary Note 5, the circuit Laplacian of the third-order reconstructed exceptional system can be written as

$$\beta^{N=3}\begin{pmatrix}V_1\\V_2\\V_3\end{pmatrix}=i\omega\begin{bmatrix}-C_{onsite} & C_1+C_2 & C_3+C_4+C_\alpha\\ C_1-C_2 & -C_{onsite} & C_1+C_2\\ C_3-C_4+C_\alpha & C_1-C_2 & -C_{onsite}\end{bmatrix}\begin{pmatrix}V_1\\V_2\\V_3\end{pmatrix}, \tag{S13}$$

where $\beta^{N=3}=i\omega(2C_1+C_3+C_4+C_\alpha)-\frac{i}{\omega L}$ and $\omega=2\pi f$. If we choose $\chi=10^{-5}$, the value of C_1, C_2, C_3, C_4, C_{onsite}, C_α and L are 23.22nF, 23.2nF, 1077.2176nF, 1077.2171nF, -1nF,

0.1*nF* and 10*uH,* respectively. Next, we multiply the left and right sides of the matrix by $-i\frac{1}{\omega}10^9$. Then, the circuit Laplacian has an excellent correspondence to that of the lattice model $H_{RES,3}$ in Eq. (1),

$$J_{N=3} = 10^9 \cdot \begin{bmatrix} -C_{onsite} & C_1 + C_2 & C_3 + C_4 + C_\alpha \\ C_1 - C_2 & -C_{onsite} & C_1 + C_2 \\ C_3 - C_4 + C_\alpha & C_1 - C_2 & -C_{onsite} \end{bmatrix}. \tag{S14}$$

Thus, we can have the relation between the frequency f and eigenvalues E_{REC} as,

$$10^9 \cdot (2C_1 + C_3 + C_4 + \alpha) - \frac{10^9}{\omega^2 L} = E_{REC}. \tag{S15}$$

When the eigenvalues are chosen as E_{REC} =22.3129, -19.313, 0, the resonance frequencies are $f = 34.051kHz, 33.824kHz, 33.925kHz$, which correspond to the results in Fig. 2(b).

We can give the resonance frequency for the sixth-order system in the same way. The circuit Laplacian for the sixth-order system can be written as

$$\beta^{N=6}\begin{pmatrix} V_1 \\ V_2 \\ V_3 \\ V_4 \\ V_5 \\ V_6 \end{pmatrix} = i\omega \begin{bmatrix} -C_{onsite} & C_1 + C_2 & C_3 + C_4 & C_5 + C_6 & C_7 + C_8 & C_9 + C_{10} + C_\alpha \\ C_1 - C_2 & -C_{onsite} & C_1 + C_2 & C_3 + C_4 & C_5 + C_6 & C_7 + C_8 \\ C_3 - C_4 & C_1 - C_2 & -C_{onsite} & C_1 + C_2 & C_3 + C_4 & C_5 + C_6 \\ C_5 - C_6 & C_3 - C_4 & C_1 - C_2 & -C_{onsite} & C_1 + C_2 & C_3 + C_4 \\ C_7 - C_8 & C_5 - C_6 & C_3 - C_4 & C_1 - C_2 & -C_{onsite} & C_1 + C_2 \\ C_9 - C_{10} + C_\alpha & C_7 - C_8 & C_5 - C_6 & C_3 - C_4 & C_1 - C_2 & -C_{onsite} \end{bmatrix} \begin{pmatrix} V_1 \\ V_2 \\ V_3 \\ V_4 \\ V_5 \\ V_6 \end{pmatrix}, \tag{S16}$$

where $\beta^{N=6} = i\omega(2C_1 + 2C_3 + C_5 + C_6 + C_7 + C_8 + C_9 + C_{10} + C_\alpha) - \frac{i}{\omega L}$. We choose the values of C_1, C_2, C_3, C_4, C_5, C_6, C_7, C_8, C_9, C_{10}, C_{onsite}, C_α and L to be 3.48*nF*, 3.33*nF*, 23.22*nF*, 23.2*nF*, 158.1155*nF*, 158.1123*nF,* 1077.2175*nF*, 1077.2171*nF*, 7338.9964*nF*, 7338.9963*nF*, -1*nF* and 10*uH,* respectively. Next, we multiply the left and right sides of the matrix by $-i\frac{1}{\omega}10^9$. Then, the circuit Laplacian has an excellent correspondence to that of the lattice model $H_{RES,6}$ in Eq. (1),

$$J_{N=6} = 10^9 \cdot \begin{bmatrix} -C_{onsite} & C_1+C_2 & C_3+C_4 & C_5+C_6 & C_7+C_8 & C_9+C_{10}+C_\alpha \\ C_1-C_2 & -C_{onsite} & C_1+C_2 & C_3+C_4 & C_5+C_6 & C_7+C_8 \\ C_3-C_4 & C_1-C_2 & -C_{onsite} & C_1+C_2 & C_3+C_4 & C_5+C_6 \\ C_5-C_6 & C_3-C_4 & C_1-C_2 & -C_{onsite} & C_1+C_2 & C_3+C_4 \\ C_7-C_8 & C_5-C_6 & C_3-C_4 & C_1-C_2 & -C_{onsite} & C_1+C_2 \\ C_9-C_{10}+C_\alpha & C_7-C_8 & C_5-C_6 & C_3-C_4 & C_1-C_2 & -C_{onsite} \end{bmatrix}. \quad \text{(S17)}$$

Therefore, we can obtain the relation between the frequency f and eigenvalues E_{REC} as:

$$10^9 \cdot \left(2C_1 + 2C_3 + C_5 + C_6 + C_7 + C_8 + C_9 + C_{10} + C_\alpha\right) - \frac{10^9}{\omega^2 L} = E_{REC}. \quad \text{(S18)}$$

When E_{REC} =57.26, -51.26, 0, the resonance frequency $f = 12.155kHz, 12.117kHz, 12.135kHz$, which correspond to the results in Fig. 2(c).

Supplementary Note 8. The sensitivity of the second-, fourth-and fifth-order RECs, and the comparison of sensitivity between the RECs and EP circuits.

In this section, we provide a detailed description of sensitivity of the second-, fourth-and fifth-order RECs. The voltage distribution in the second-, fourth-and fifth-order systems are addressed below in Supporting Figure 5. The red, blue, and green lines represent the voltage distributions when the perturbation capacitor C_α = 0.1, 0.01 and 0.001*nF*, respectively. It can be clearly seen that the splitting of resonance frequency increases with the increase of disturbance. It corresponds to the theoretical results in Fig. 1(c).

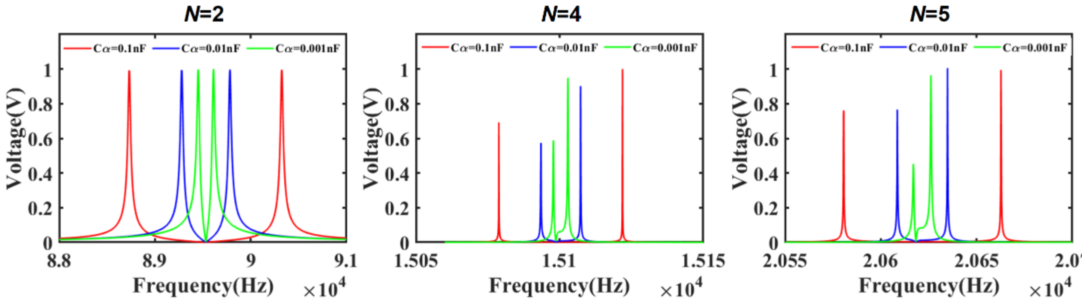

Supporting Figure 5. The simulated voltage distributions when C_α = 0.1, 0.01 and 0.001*nF* in the second-, fourth-and fifth-order RECs.

Based on the splitting of resonance frequency, in Supporting Figure 6, we show the sensitivity in the second-, fourth-and fifth-order RECs. The red, blue, and black dots represent

the simulated sensitivities of the second-, fourth-and fifth-order RECs, while the blue, red and yellow lines represent the theoretical sensitivities of second-, fourth-and fifth-order systems. We find that the simulated results agree with the theoretical results very well. The high agreement means that the ultra-sensitivity can be realized in our designed circuit.

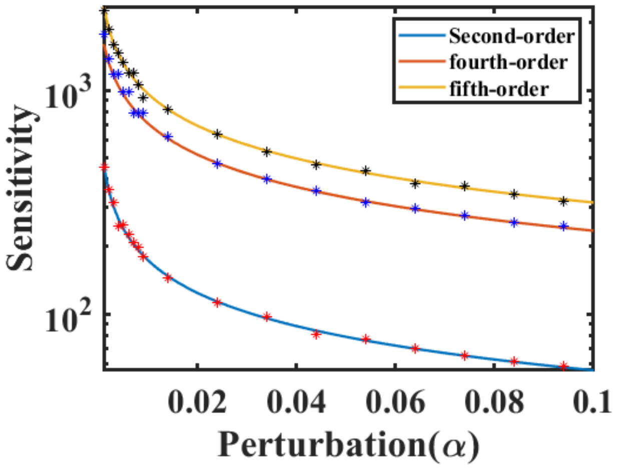

Supporting Figure 6. Comparisons between theoretical and simulated results for the sensitivity in the second-, fourth-and fifth-order RECs.

More importantly, the sensitivity for the REC is better than that of the EP circuit. In the following, we give the details of simulation results for the sixth-order REC and EP circuits. The energy difference and sensitivity for the REC and EP circuit are shown in Supporting Figure 7(a) and 7(b), respectively. The solid red and blue lines are the analytical results, and the corresponding dots are simulated results. It is clearly seen that in the range from $3pF$ to $200pF$ the sensitivity for the REC displays about two orders of magnitude larger than that of the EP circuit. It means that for a fixed C_α, the resonance frequency variation of the REC is significant, much larger than that of the EP circuit. Therefore, instead of the EP circuit, it is more advantageous to use the REC in the detection of small perturbations.

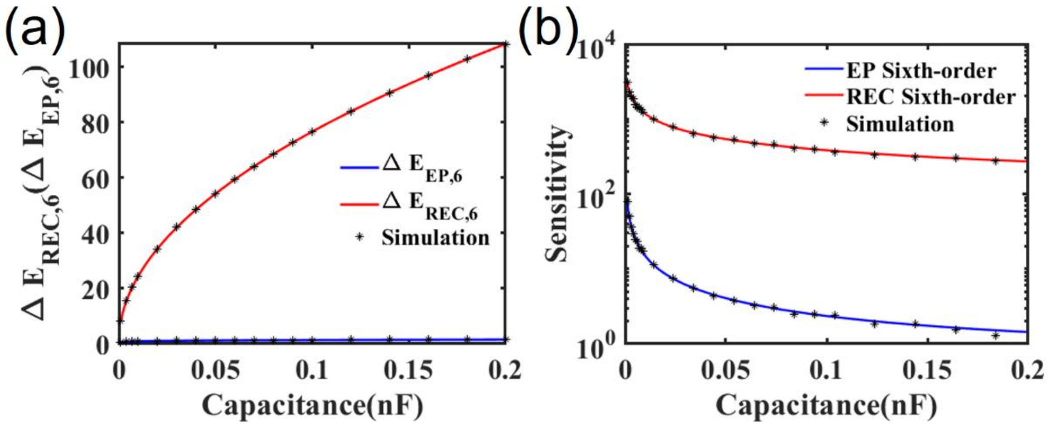

Supporting Figure 7. The energy difference and sensitivity for the sixth-order REC and EP circuit. Blue solid lines represent the results for the sixth-order EP circuit and red solid lines denote the results for the sixth-order REC. The simulation results are shown in black dots.

Supplementary Note 9. Circuit fabrications and measurements in experiment.

In this part, we give details on the circuit fabrications and measurement in experiment.

(a) *The selection of circuit elements*. We note that the resistance within components can introduce non-Hermitian losses in the experiment. For a single capacitive, resistive and inductive component, the current is respectively proportional to $\mathrm{i}\omega C$, $1/R$, and $1/\mathrm{i}\omega L$ times the voltage across it, with the listed coefficients being the effective admittance (inverse impedance). While energy is lost, the voltage and currents can be maintained. Our circuit contains many more components, but analogously, the non-Hermitian admittance only at most amounts to phase shifts between the current and voltage. Energy loss is expected in the circuit and they are already built into the formalism.

While the circuit Laplacian's eigenvalues are already computed based on the losses within the formalism, let us also discuss what happens if there are (inevitable) losses associated with the circuit components i.e. parasitic resistances. For instance, in Eq. (S10), we can replace $\mathrm{i}\omega L$ with $\mathrm{i}\omega L + R_L$, where R_L represents the inductive loss. In our experiment, we choose a small R_L, the eigenvalues will not be substantially affected, and the signals will undergo a small phase shift. And correspondingly the height of the voltage peak in Fig. 3(c) of the main text will be affected, since the resonance peaks will be broadened by this quantity. However, the

R_L we chose was small enough that the voltage distribution did not significantly change our results. Therefore, it is vital that the applied inductor should have a high Q value at the operational frequency.

Here, we chose MWSA0603S-100MT of inductors, and coupling capacitors of the C0G material are used to match the operational frequency range. The Operational Amplifiers (LM6171) are used to construct voltage follower modules. The resistor R_α (2000Ω) and the capacitor C_α (1000pF) can work as a negative feedback network, which plays a key role in improving the load capacity of the voltage follower and ensures the nearly identical values for the input and output voltages.

(b) *Sample fabrications*. Firstly, the capacitances with different values are hard to find in market. So, we buy capacitances with 10% error to select the capacitances we need. After that, we exploit the electric circuits by using PADs program software, where the PCB composition, stackup layout, internal layer and grounding design are suitably engineered. It is worthy to note that the ground layer should be placed in the gap between any two layers to avoid their coupling. Moreover, all PCB traces have a relatively large width (0.5mm) to reduce the parasitic inductance and the spacing between electronic devices is also large enough (1.0mm) to avert spurious inductive coupling. Voltage follower is constructed with the help of opamp, for which the model is LM6171. Besides, we use a Pin header to measure the voltage at various nodes. Supporting Figure 8 shows the photograph image for sixth-order system. The purple dashed box displays the magnified portion between two nodes, where the blue rectangle represents the circuit construction of the voltage follower, which includes the operational amplifier (Tools Instrument, LM6171) and the resistor R_α (2000 Ω) and the capacitor C_a (1000pF) work as a negative feedback network. The green and red boxes form the effective non-reciprocal capacitance value.

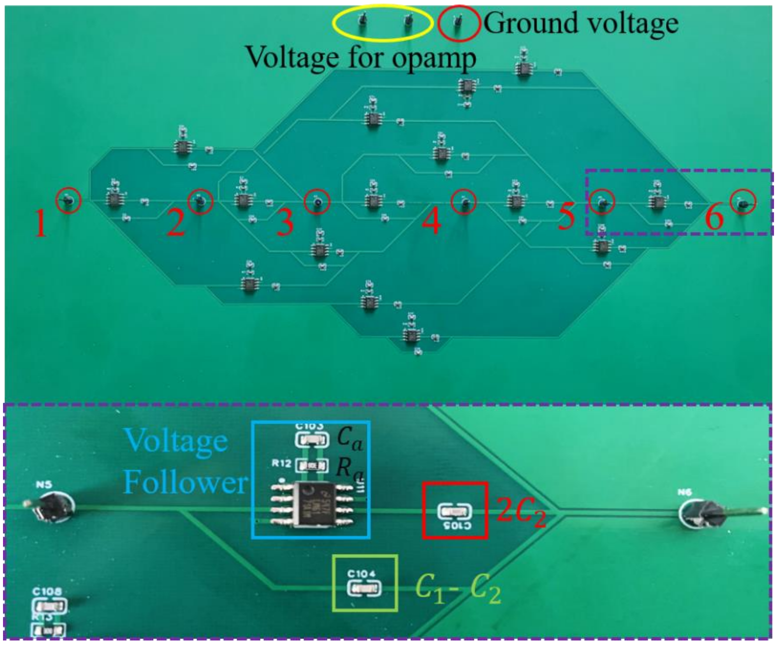

Supporting Figure 8. The photograph image for the sixth-order RECs.

(c) *Circuit measurements*. For such circuit, we use DC power supply (UNI-T UTP1306S) to generate $\pm 3.5V$ DC voltages for opamps. We use the arbitrary/function waveform generator (ROGOL DG1022Z) to create the required AC voltage signal with amplitude 2V. For the convenience of experiment, we input the voltage on node 1. Then, the digital storage oscilloscope (Agilent Technologies Infiniivision DSO7104B) is used to measure the voltage signal at node 1 simultaneously.

Supplementary Note 10. Experimental voltage distribution voltage distributions and sensitivities for the other orders.

In this part, we provide the experimental voltage distribution in the system with N=2, 4, 5, 6. The red, blue, and green lines in Supporting Figure 9 represent the voltage distributions when the perturbation capacitor C_α = 0.1, 0.01 and 0.001nF, respectively. It can be clearly seen that the splitting of resonance frequency increases with the increase of perturbation, which corresponds to the theoretical results in Fig. 2(b).

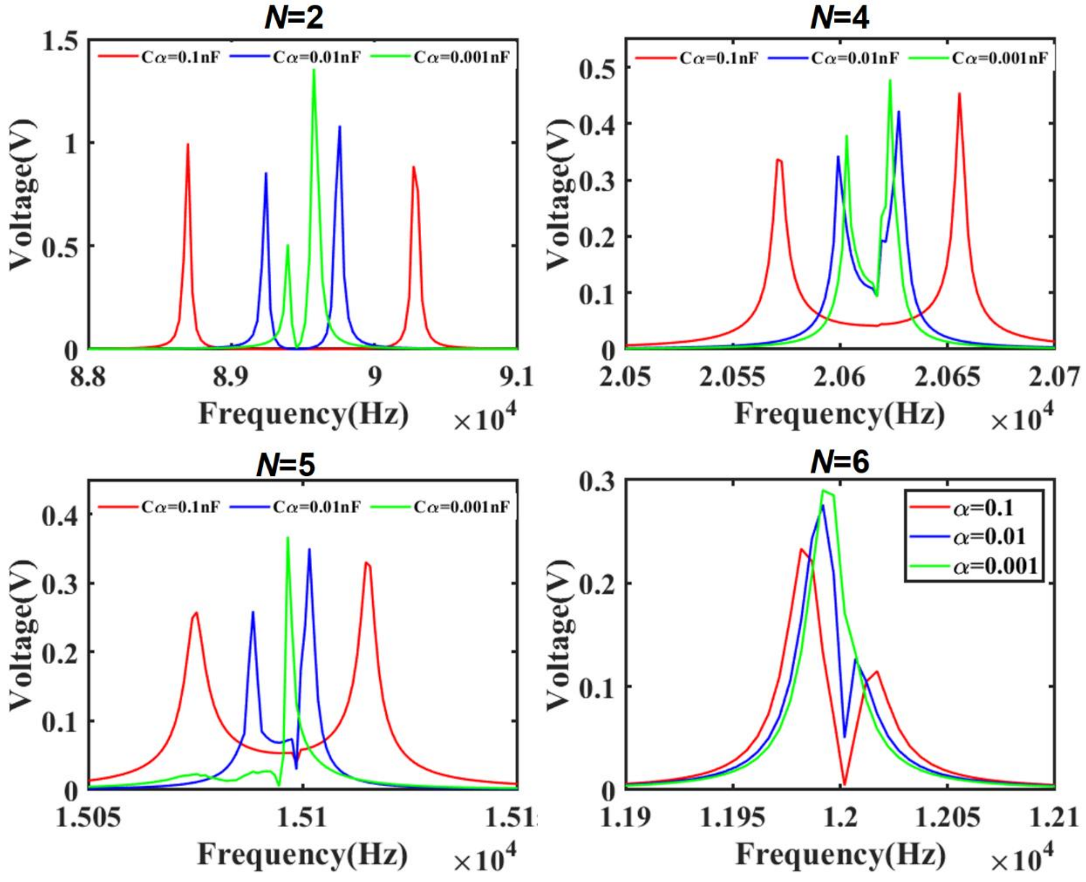

Supporting Figure 9. The voltage distributions when $C_\alpha = 0.1$, 0.01 and $0.001nF$ in the RECs with *N*=2, 4, 5, 6**.**

In the following, we show the experimental sensitivity of second-, fourth-and fifth-order reconstructed exponential circuits in Supporting Figure 10. The red, blue, and black dots represent the experimental sensitivity of second-, fourth-, and fifth-order RECs, respectively. The blue, red, and yellow lines represent the theoretical sensitivities of the second-, fourth-and fifth-order RECs, respectively. We find that the experimental results correspond well with the theoretical results. The high agreement means that the ultra-sensitive sensor is successfully realized in our circuit.

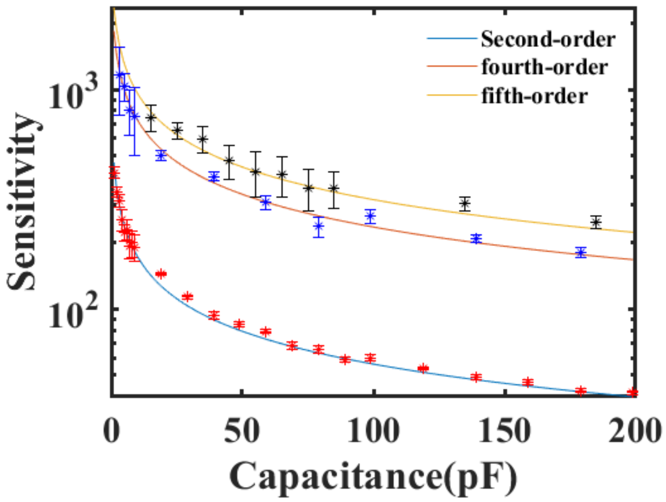

Supporting Figure 10. Comparisons between theoretical and experimental results for sensitivity in the system with N=2, 4 and 5.

Supplementary Note 11. The fabrication of displacement capacitor.

In this section, we provide a detailed introduction to the fabrication of the displacement capacitor. The displacement capacitors can be equivalent to the parallel-plate capacitor. Specifically, the displacement capacitance is calculated as $C_d = \varepsilon_a wd / s$, where ε_a is the permittivity of air, w is the width of rectangular electrodes, d is the transverse displacement, and s is the spacing of electrodes. The displacement capacitance in Fig. 3(b) consists of two parallel rectangular electrodes with the copper foil which are fixed on the displacement stage. Here, the change of distance d is achieved by tuning the knob for transverse motion of Electrode 1 in the displacement capacitor. The change of spacing of electrodes s is achieved by tuning the knob for vertical motion in the displacement capacitor. To achieve the displacement capacitors with 1-200pF, we firstly adjust the size of electrodes and the knob for the change of spacing of electrodes s to determine the initial and final value of displacement capacitors. Then, by tuning the knob for transverse motion of distance d, we achieve the displacement capacitors with 1-200pF.

Supplementary Note 12. The analysis of signal-to-noise ratio in the reconstructed exponential circuit.

In this part, we give the noise analysis of reconstructed exponential circuits. The previous

studies have pointed out that the influence of noises on the designed sensors imposes the fundamental bound on the sensitivity. In this case, the influence of noises should be clarified in our designed circuit sensors. In the experiment, there are two main sources of noise in the measurement of frequency: a) The parasitic errors of coupling devices in sensing systems, such as parasitic resistance. b) Thermal noise in active devices.

For the parasitic errors, we note that the loss of inductance significantly influences the value and width of the impedance peak for resonance frequency. Therefore, we choose the inductances with a high Q value at the operational frequency in experiment. Besides, to ensure accurate experimental implementation of our theoretically designed circuit Laplacian, the component uncertainties have to be minimized and their stability optimized. Capacitances and inductances with 0.5% error tolerance are obtained by selecting from a large sample of commercially available capacitances with 10% error tolerance. With the control of error tolerance, the impact of the parasitic errors on our system becomes very small, which is shown by error bar in Fig. 3(d). In Supplementary Note 9, we give details on the selection of circuit elements.

For the thermal noise in active devices, we divide the frequency range into several areas. After scanning a frequency area, we wait for the system to cool down before scanning the next area. Due to the small scanning area and short working time, the thermal noise is effectively suppressed. When the order of our system increases, the parasitic and thermal errors are effectively suppressed by the control of error tolerance and reasonable measurement method. So, the signal-to-noise ratio of the circuit we designed is very stable and does not increase with the increase of order.

To measure thermal noise in the experiment, we input a 20kHz AC signal into node1 and then perform Fourier analysis on the electrical signal of measurement point 1. As shown in Supporting Figure 11(a), we can see that the signal is mainly concentrated at 20kHz, and the signal distribution at other frequencies is mainly affected by noise. By comparing the input frequency and noise frequency, we obtained the circuit signal-to-noise ratio, as shown in Supporting Figure 11(b). The figure shows the signal-to-noise ratio of the second- to sixth-order circuit, and we can see that the signal-to-noise ratio of the circuit is very stable, basically around

9. And it will not increase with the increase of circuit order. This fully demonstrates the stability of our circuit.

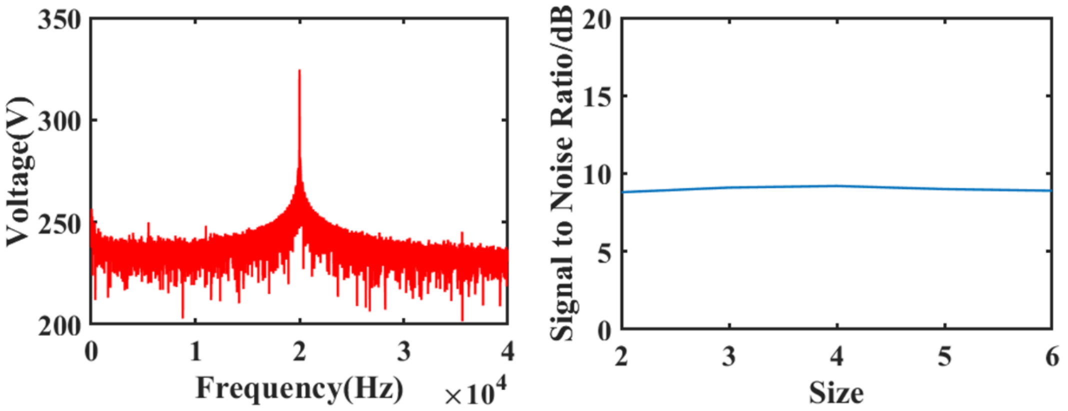

Supporting Figure 11. (a) Signal frequency analysis results. (b)The results for signal to noise ratio in RECs with N=2-6.

Supplementary Note 13. The effect of long-range couplings in the sensing.

In this discussion above, we have proposed the RES and show the performance as an ultra-sensitive circuit sensor. It shows the comprehensive improvement when compared with the high-order EP sensor. As shown in the construction of RES (the first part of Results section in the main text), the couplings of $H_{\mathrm{RES},N}$ not only exist in the nearest neighboring lattices, but also among the two distant lattices. The coupling strengths and the length of the chain exhibit a power-law relationship. Here, we will show the effect of long-range couplings on the sensor performance. The Hamiltonian for the N-order RES is

$$H_{\mathrm{RES},N}=\begin{pmatrix} 1 & \chi^{-\frac{1}{N}} & \cdots & \chi^{-\frac{N-1}{N}} \\ \chi^{\frac{1}{N}} & 1 & \cdots & \chi^{-\frac{N-2}{N}} \\ \vdots & \vdots & \ddots & \vdots \\ \chi^{\frac{N-1}{N}} & \cdots & \chi^{\frac{1}{N}} & 1 \end{pmatrix}_{N\times N} \tag{S19}$$

When removing the long-range couplings, the system Hamiltonian in Eq. (S19) changes to

$$H'_N = \begin{pmatrix} 1 & \chi^{-\frac{1}{N}} & 0 & \cdots & 0 \\ 0 & 1 & \chi^{-\frac{1}{N}} & 0 & \vdots \\ \vdots & \vdots & \ddots & \vdots & 0 \\ 0 & 0 & \cdots & 1 & \chi^{-\frac{1}{N}} \\ 0 & 0 & \cdots & 0 & 1 \end{pmatrix}_{N\times N} \quad \text{(S20)}$$

The diagonal elements of the Hamiltonian H'_N are 1, and only the nearest couplings are kept which are set as $\chi^{-\frac{1}{N}}$. When comparing with the EP Hamiltonian (Eq. (S1) in Supplementary Note 1), the off-diagonal elements in Eq. (S20) are $\chi^{-\frac{1}{N}}$. In our study, the sensed term displays the connection between the first and last lattices. When the perturbation strength induced by the sensed item is α, the Hamiltonian for the sensed term is $H_{p,N} = \begin{pmatrix} 0 & \cdots & 0 & \alpha \\ \vdots & \ddots & \vdots & \vdots \\ 0 & \cdots & \ddots & 0 \\ \alpha & 0 & \cdots & 0 \end{pmatrix}_{N\times N}$. In the following, we will give the study of sensor performance with the order $N=5$ and $N=6$.

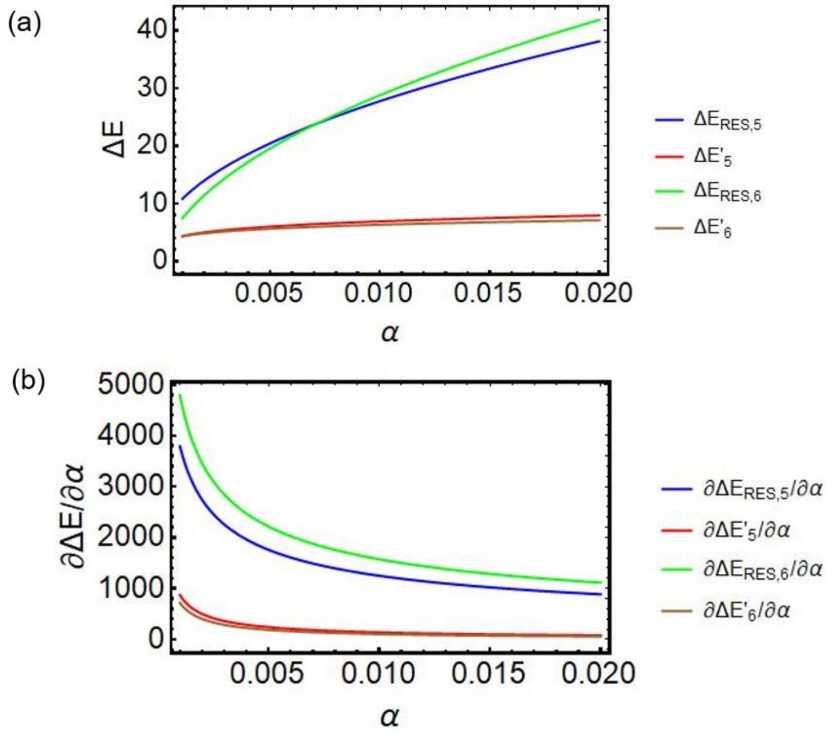

Supporting Figure 12. (a) The relationship between energy difference and the perturbation strength, for the fifth-order (blue solid line) and the sixth-order (green solid line) RES, and

H'_N with $N=5$ (red solid line) and $N=6$ (brown solid line). (b) The sensitivity $\frac{\partial \Delta E_{\mathrm{RES},N}}{\partial \alpha}$ ($\frac{\partial \Delta E'_N}{\partial \alpha}$) with the perturbation strength α. The blue and green solid lines correspond to the RES with $N=5$ and $N=6$. The red and brown solid lines correspond to H'_N with $N=5$ and $N=6$. For all panels, the parameter $\chi=10^{-6}$.

From Supporting Figure 12(a), it is clearly seen that the energy difference $\Delta E_{\mathrm{RES},N}$ between the neighboring eigenstates in the reconstructed exceptional system exhibits one orders of magnitude larger than that in the system with Hamiltonian H'_N. In addition, the sensitivity results are also provided. In Supporting Figure 12(b), we provide the sensitivity for the reconstructed exceptional system with the order $N=5$ and $N=6$, and the corresponding results are shown in blue, and green solid lines. When compared with the sensitivity in the system with Hamiltonian H'_N, its sensitivity (blue and green solid line) also displays one order of magnitude larger than that of H'_N (red and brown line).

Supplementary Note 14. The robustness of the REC.

In this section, we analyze the robustness of our designed circuit. We take the results for the circuit with *N*=3 and 6 when $C_\alpha = 0.004nF, 0.01nF, 0.05nF, 0.1nF$ and $0.2nF$ as an example. We use capacitors and inductors with a tolerance of 10% to simulate the theoretically predicted uncertainty of the resonant frequency. Next, the energy difference ΔE_{REC} based on the resonance frequencies are obtained. The results for the circuit with *N*=3 and 6 are shown in Supporting Figure 13(a) and (b), respectively. The blue and red solid lines are the analytical results for the circuit with *N*=3 and 6, respectively. The black dots are simulated results using LTspice. The green lines are simulated uncertainty of ΔE_{REC}. When adding the error tolerance, it is seen that the largest deviation with the analytical results is within the range [-2, 1.4] and [-7, 7] for the circuit with *N*=3 and 6, respectively. Therefore, the deviation in the ΔE_{REC} is about 6%. Because the variation of energy difference is rather small when the disorder is

considered, the ultra-sensitivity ($\frac{\partial \Delta E_{\mathrm{REC},N}}{\partial \alpha}$) can still be remarkable for it relates to the differential relation with perturbation amplitude α . In this way, it indicates the robustness of the enhanced sensitivity in our designed circuit. Moreover, since the enhanced sensitivity can be revealed even with such large disorder, it is also believed that our RES can also achieved in other classical systems.

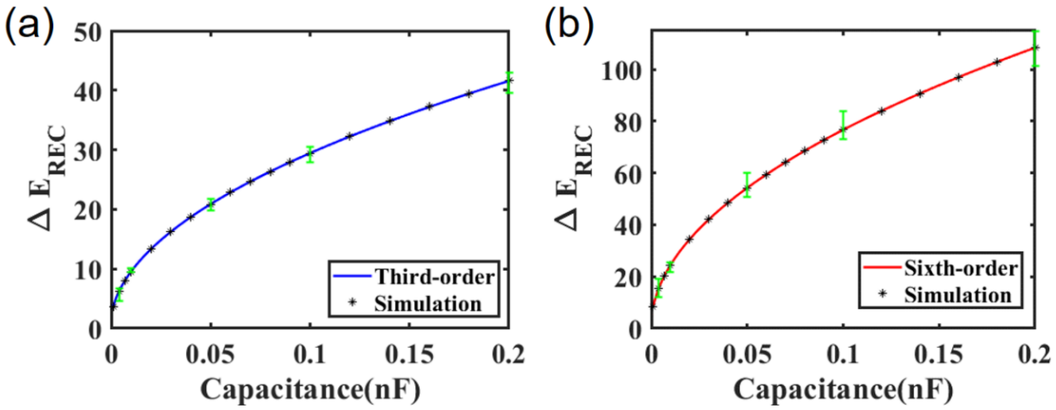

Supporting Figure 13. The uncertainty of energy difference for the third-order and sixth-order REC. The blue and red solid lines are the analytical results for the circuit with N=3 and 6, respectively. The black dots are simulated results using LTspice. The green lines are simulated uncertainty of ΔE_{REC} .
